# Urolithin Α modulates inter-organellar communication via calcium-dependent mitophagy to promote healthy ageing

**DOI:** 10.1080/15548627.2025.2561073

**Published:** 2025-09-29

**Authors:** Antonis Roussos, Katerina Kitopoulou, Fivos Borbolis, Christina Ploumi, Despoina D. Gianniou, Zhiquan Li, Haijun He, Eleni Tsakiri, Helena Borland, Ioannis K. Kostakis, Martina Samiotaki, Ioannis P. Trougakos, Vilhelm A. Bohr, Konstantinos Palikaras

**Affiliations:** a Department of Physiology, Medical School, National and Kapodistrian University of Athens, Athens, Greece; b Department of Biology, National and Kapodistrian University of Athens, Athens, Greece; c Danish Center for Healthy Aging, Department of Cellular and Molecular Medicine, Faculty of Health and Medical Sciences, University of Copenhagen, Copenhagen, Denmark; d Department of Pharmacy, Division of Pharmaceutical Chemistry, National and National and Kapodistrian University of Athens, Athens, Greece; e Biomedical Sciences Research Center “Alexander Fleming”, Institute for Bioinnovation, Vari, Greece

**Keywords:** Calcium, cellular senescence, ER, geroprotection, lysosome, mitochondria

## Abstract

Mitochondrial dysfunction and impaired mitophagy are hallmarks of ageing and age-related pathologies. Disrupted inter-organellar communication among mitochondria, endoplasmic reticulum (ER), and lysosomes, further contributes to cellular dysfunction. While mitophagy has emerged as a promising target for neuroprotection and geroprotection, its potential to restore age-associated defects in organellar crosstalk remains unclear. Here, we show that mitophagy deficiency deregulates the morphology and homeostasis of mitochondria, ER and lysosomes, mirroring age-related alterations. In contrast, Urolithin A (UA), a gut-derived metabolite and potent mitophagy inducer, restores inter-organellar communication via calcium signaling, thereby, promoting mitophagy, healthspan and longevity. Our multi-omic analysis reveals that UA reorganizes ER, mitochondrial and lysosomal networks, linking inter-organellar dynamics to mitochondrial quality control. In *Caenorhabditis elegans*, UA induces calcium release from the ER, enhances lysosomal activity, and drives DRP-1/DNM1L/DRP1-mediated mitochondrial fission, culminating in efficient mitophagy. Calcium chelation abolishes UA-induced mitophagy, blocking its beneficial impact on muscle function and lifespan, underscoring the critical role of calcium signaling in UA’s geroprotective effects. Furthermore, UA-induced calcium elevation activates mitochondrial biogenesis via UNC-43/CAMK2D and SKN-1/NFE2L2/Nrf2 pathways, which are both essential for healthspan and lifespan extension. Similarly, in mammalian cells, UA increases intracellular calcium, enhances mitophagy and mitochondrial metabolism, and mitigates stress-induced senescence in a calcium-dependent manner. Our findings uncover a conserved mechanism by which UA-induced mitophagy restores inter-organellar communication, supporting cellular homeostasis and organismal health.

**Abbreviations**: Ca^2+^: calcium ions; BJ: human foreskin fibroblasts; BNIP3: BCL2 interacting protein 3; BP: bipyridyl; CAMK2D: calcium/calmodulin dependent protein kinase II delta; CCCP: carbonyl cyanide m-chlorophenyl hydrazone; DEGs: differentially expressed genes; DEPs : differentially expressed peptides; DFP: deferiprone; DNM1L/DRP1: dynamin 1 like; EGTA: ethylene glycol bis(2-aminoethyl ether)-N,N,N’,N’-tetraacetic acid; EMC: endoplasmic reticulum membrane protein complex; ER: endoplasmic reticulum; FCCP: carbonyl cyanide p-trifluoro-methoxyphenyl hydrazone; GO: gene ontology; GSVA: Gene Set Variation Analysis; HUVECs: human umbilical vein endothelial cells; IMM: inner mitochondrial membrane; ITPR/InsP3R: inositol 1,4,5-triphosphate receptor; MAM: mitochondria-associated ER membrane; MAPK: mitogen-activated protein kinase; MCU: mitochondrial calcium uniporter; MEFs: mouse embryonic fibroblasts; NAC : N-acetylcysteine; NFE2L2/Nrf2: NFE2 like bZIP transcription factor 2; NMN: nicotinamide mononucleotide; NR: nicotinamide riboside; OMM: outer mitochondrial membrane; PCA: principal-component analysis; PPARGC1A/PGC1α: PPARG coactivator 1 alpha; PQ: paraquat; TMCO: transmembrane and coiled-coil domains 1; TMRE: tetramethylrhodamine ethyl ester perchlorate; UA: urolithin A; VDAC: voltage dependent anion channel.

## Introduction

Ageing is characterized by a progressive deterioration of cellular function and structural integrity, driven by the breakdown of key homeostatic pathways [1,2]. Among these, mitochondrial dysfunction and impaired mitochondrial selective autophagy (known as mitophagy) are established hallmarks of ageing and age-associated pathologies, including neurodegeneration and metabolic disorders [1,2,3–6]. While mitochondria have traditionally been considered as autonomous energy-producing organelles, recent studies highlight their dynamic communication with other membrane-bound compartments, particularly the endoplasmic reticulum (ER), peroxisomes and lysosomes [7–10]. This inter-organellar communication coordinates critical cellular processes, including calcium signaling, lipid trafficking, energy metabolism, and the maintenance of organelle integrity [6,7,11–13].

Emerging evidence suggests that disrupted inter-organellar crosstalk is a pivotal but underappreciated contributor to cellular ageing [6,11–14]. However, the upstream regulators of this process and whether it can be restored remain poorly understood. Here, we uncover that mitophagy deficiency phenocopies age-associated defects in organelle morphology, including fragmented and dysfunctional mitochondria, distorted ER architecture, and compromised lysosomal integrity. These findings indicate that mitophagy is not only essential for mitochondrial turnover but also for the maintenance of broader organelle organization and communication.

Pharmacological modulation of mitophagy presents a promising therapeutic strategy to tackle mitochondrial and age-related diseases [15–18]. Among various natural compounds identified as mitophagy inducers, Urolithin A (UA) stands out due to its safety profile and its conserved ability to stimulate mitophagy across several model organisms, from the nematode *Caenorhabditis elegans* to humans [19–22]. UA, a metabolite derived from the gut microbial transformation of ellagitannins, has been shown to enhance muscle function, prevent the accumulation of dysfunctional mitochondria with age and extend lifespan in *C. elegans* [4,19]. Extensive studies in mammalian cells, nematodes and mouse models have demonstrated that UA promotes cellular and tissue homeostasis primarily through mitophagy induction [4,19,20,23,24]. Notably, the beneficial effects of UA are conserved in humans, with UA supplementation having received approval from the U.S. Food and Drug Administration (FDA) as a safe ingredient for food products and dietary supplements [25–27]. These findings highlight the therapeutic potential of UA as a novel intervention to improve mitochondrial function and counteract age-dependent comorbidities [28]. However, the mechanism by which UA influences cellular homeostasis remains incompletely understood, particularly beyond its effects on mitochondrial turnover.

We report that UA-induced mitophagy restores age-associated organellar defects and reorganizes ER-mitochondria-lysosome communication through calcium signaling. UA supplementation elevates intracellular calcium levels in various cell types promoting mitophagy both in nematodes and mammalian cells. In *C. elegans*, UA triggers ER calcium release via ITR-1/ITPR/InsP3R (inositol 1,4,5-triphosphate receptor), TMCO-1/F22B5.10/TMCO1 (transmembrane and coiled-coil domains 1), and EMC-3/EMC (ER membrane protein complex subunit 3). The released calcium ions (Ca^2+^) enhance lysosomal function and are taken up by mitochondria through MCU-1/MCU (mitochondrial calcium uniporter), facilitating DRP-1/DNM1L/DRP1 (dynamin-related protein 1)-mediated mitochondrial fission (a critical step for mitophagy). UA also mediates mitochondrial biogenesis through the UNC-43/CaMK2D (calcium/calmodulin dependent protein kinase II delta)-SKN-1/NFE2L2/Nrf2 (NFE2 like bZIP transcription factor 2) pathway, thereby supporting energy metabolism and promoting healthspan and lifespan. Disruption of calcium elevation inhibits UA-induced mitophagy, impairs muscle function and diminishes lifespan extension in *C. elegans*. In human cells, UA supplementation not only triggers DRP1-mediated mitophagy but also enhances mitochondrial metabolism, lysosomal activity and mitigates stress-induced cellular senescence in a calcium-dependent manner. Together, our findings uncover an evolutionarily conserved mechanism by which mitophagy preserves organelle architecture and inter-organellar communication, positioning UA as a therapeutic candidate for restoring intracellular homeostasis, improving energy metabolism and normal organismal physiology during ageing.

## Results

### Mitophagy deficiency phenocopies age-associated organellar morphology defects in *C. elegans*

Disruption of organelle interactions and inter-communication is increasingly recognized as a key contributor to cellular ageing and age-related diseases [7,11–13]. However, the precise regulatory mechanisms governing organellar crosstalk, particularly during ageing, remain poorly understood. To investigate whether mitophagy influences organellar architecture during ageing, we assessed the morphology of ER, mitochondria, and lysosomes in *C. elegans* using organelle-specific markers. Confocal microscopy revealed significant age-dependent deterioration in organelle morphology at day 8 compared to day 1 wild-type control nematodes (Figure S1A-C). Specifically, aged nematodes exhibited fragmented, aggregated and swollen ER structures, disorganized, globular and fragmented mitochondrial networks, enlarged and filamentous lysosomes, indicating impaired organelles dynamics and homeostasis. Interestingly, knocking down key mitophagy regulators, including PINK-1, PDR-1, and DCT-1 (the nematode homologs of the mammalian PINK1, PRKN/parkin and BNIP3 (BCL2 interacting protein 3) respectively), exacerbated the age-related changes in organelle morphology, accelerating defects in mitochondrial and lysosomal structures. Consistently, young (day 1) nematodes subjected to RNAi against mitophagy genes displayed filamentous ER, disrupted mitochondrial network, and enlarged lysosomes, closely resembling organellar phenotypes observed in aged controls (Figure S1A-C).

Over the last decade, several natural and synthetic compounds have been identified as potent mitophagy stimulators [16,18]. These mitophagy inducers include general toxicants such as rotenone, paraquat (PQ), carbonyl cyanide m-chlorophenyl hydrazone (CCCP) and carbonyl cyanide p-trifluoro-methoxyphenyl hydrazone (FCCP), iron chelators like deferiprone (DFP) and bipyridyl (BP), NAD^+^ precursors including nicotinamide riboside (NR) and nicotinamide mononucleotide (NMN) as well as spermidine, resveratrol, and antibiotics such as valinomycin, salinomycin, and antimycin A [16,18]. Among these, UA stands out as one of the most potent mitophagy inducers. Despite our emerging understanding of the molecular mechanisms through which compounds like iron chelators, NAD^+^ boosters, general toxicants and antibiotics trigger mitophagy, the mechanisms by which UA induces mitophagy remain largely elusive, representing a crucial gap in our understanding of its therapeutic potential. Remarkably, UA treatment restored normal organelle morphology even in aged worms (Figure S1A-C). UA-treated animals showed significant restoration of ER network structure, improved mitochondrial integrity, and normalized lysosomal morphology and distribution (Figure S1A-C).

**Figure 1.**
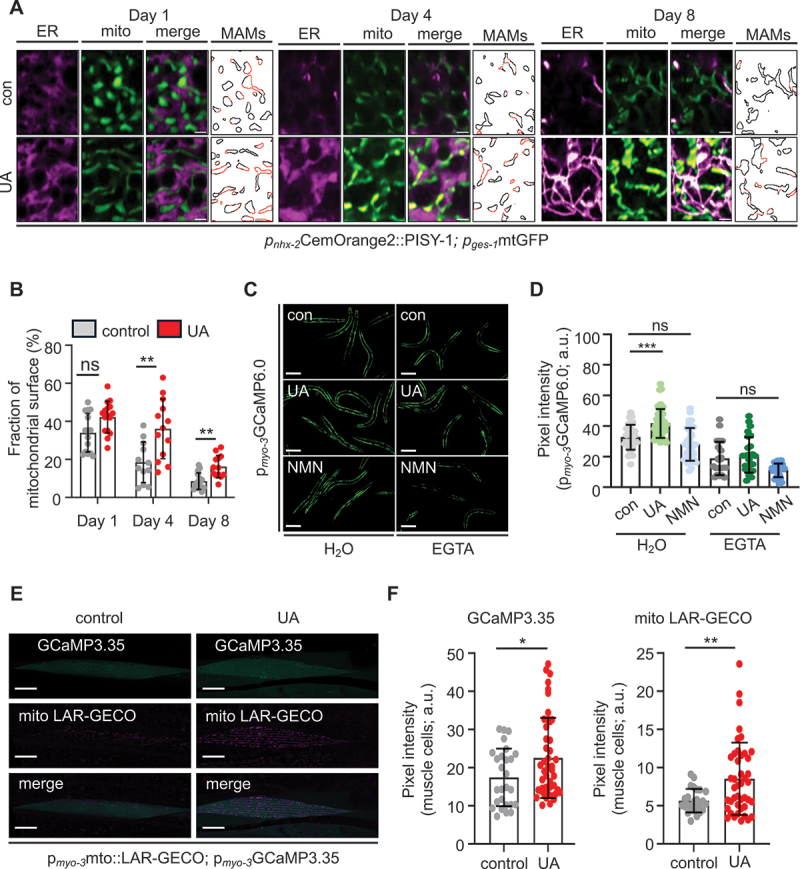
Urolithin A ameliorates the age-dependent decline of ER-mitochondria contacts and alters intracellular Ca^2+^ balance. (A and B) Representative images (A) and quantification of MAMs (B) during ageing of nematodes co-expressing an ER-localized CemOrange (magenta) and a mitochondria-targeted GFP (green) in their intestine (*nhx-2* and *ges-1* promoters, respectively) in control conditions or treated with UA (scale bars: 2 μm; data presented as mean ± SD; ns *p* < 0.05, **p < 0.01; two-way ANOVA). The last panel in each condition in (A) depicts the surface of mitochondria. The fraction that colocalizes with the ER is marked with red and plotted in (B). (C and D) Representative images (C) and quantification (D) of fluorescence intensity in 4-day old transgenic nematodes expressing the calcium biosensor GCaMP6.0 in their body wall muscles (*myo-3* promoter), under control conditions and treated with UA or NMN, in the presence or absence (H_2_O) of the Ca^2+^-chelating agent EGTA (scale bars: 50 μm; data presented as mean ± SD; ns *p* > 0.05, ***p < 0.001; one-way ANOVA). (E and F) Representative images (E) and quantification of mitochondrial fluorescence (F) in 4-day old animals co-expressing the mitochondria-targeted LAR-GECO and the cytoplasmic GCaMP3.35 calcium biosensors in their body wall muscles (*myo-3* promoter) (scale bars: 20 μm; data presented as mean ± SD; *p < 0.05, **p < 0.01; unpaired *t-t*est).

To further investigate how mitophagy induction impacts inter-organellar communication, we examined mitochondria-associated ER membranes (MAMs), which are specialized contact sites critical for inter-organellar signaling. Using high-resolution confocal microscopy, we observed an age-dependent reduction in MAMs formation from day 1 to day 8 in wild-type nematodes, suggesting compromised inter-organellar communication with increasing age (Figure 1A,B). Notably, UA supplementation significantly increased MAMs abundance, restoring their formation in aged animals (Figure 1A,B). Quantitative analysis confirmed a significant increase in MAMs upon UA treatment compared to aged untreated animals, highlighting the ability of UA to restore inter-organellar connectivity (Figure 1B).

Taken together, these findings demonstrate that impaired mitophagy in young nematodes recapitulates key age-related disruptions in organelle architecture and inter-organellar communication, while UA-induced mitophagy effectively reverses these changes, underscoring its therapeutic potential for promoting cellular health during ageing.

### UA supplementation increases intracellular calcium levels to promote mitophagy in *C. elegans*

Intracellular calcium levels play a pivotal role in cellular homeostasis, as Ca^2+^ act as versatile second messengers influencing a wide array of cellular processes, including energy metabolism, cell differentiation and survival. Moreover, the ability of calcium to modulate enzymatic activities is crucial, particularly under stress conditions [14, 29]. Previous studies in nematodes have shown that PQ and heat shock lead to cytosolic calcium increase in neurons and muscles [30–32]. Therefore, we used PQ as a positive control and investigated whether other mitophagy-inducing compounds, such as UA and NMN, similarly affect intracellular calcium levels. To monitor cytosolic calcium levels, we treated transgenic nematodes expressing the GCaMP2.0 calcium biosensor under the ubiquitous promoter *let-858*, with UA, PQ or NMN. As expected, PQ administration increased the fluorescent intensity of the GCaMP2.0 biosensor, indicating elevated calcium levels. Interestingly, UA also produced a similar increase in calcium levels (Figure S2A, B). Furthermore, both UA and PQ treatments enhanced calcium levels in muscle and neuronal cells (Figure 1C,D and Figure S2C, D). Notably, NMN did not influence cytosolic calcium in any tissue examined (Figure 1C,D and Figure S2A-D). To validate our experimental setup, we used the calcium chelating agent EGTA (ethylene glycol bis (2-aminoethyl ether)-N,N,N’,N’-tetraacetic acid) and found that it inhibited both UA- and PQ-induced calcium elevation in all examined tissues (Figure 1C,D and Figure S2A-D).

The proximity between ER and mitochondria facilitates the establishment of high-concentration Ca^2+^ microdomains, which promote rapid mitochondrial calcium uptake [14]. Given that UA enhances ER-mitochondria interactions, at early life stages and prevents their age-dependent dissociation (Figure 1A,B), we examined whether mitochondrial calcium levels are also influenced by UA supplementation. To evaluate mitochondrial calcium levels, we used transgenic nematodes co-expressing the mitochondrial calcium biosensor mito::LAR-GECO and the cytosolic calcium biosensor GCaMP3.35 in muscle cells [33]. Notably, UA administration significantly elevated fluorescence intensities of both biosensors, confirming increased calcium levels in the cytosol and within mitochondria (Figure 1E,F). These results suggest that UA treatment enhances ER-mitochondria contacts facilitating calcium redistribution, which then potentially initiates a calcium-dependent signaling cascade that regulates mitochondrial quality control.

**Figure 2.**
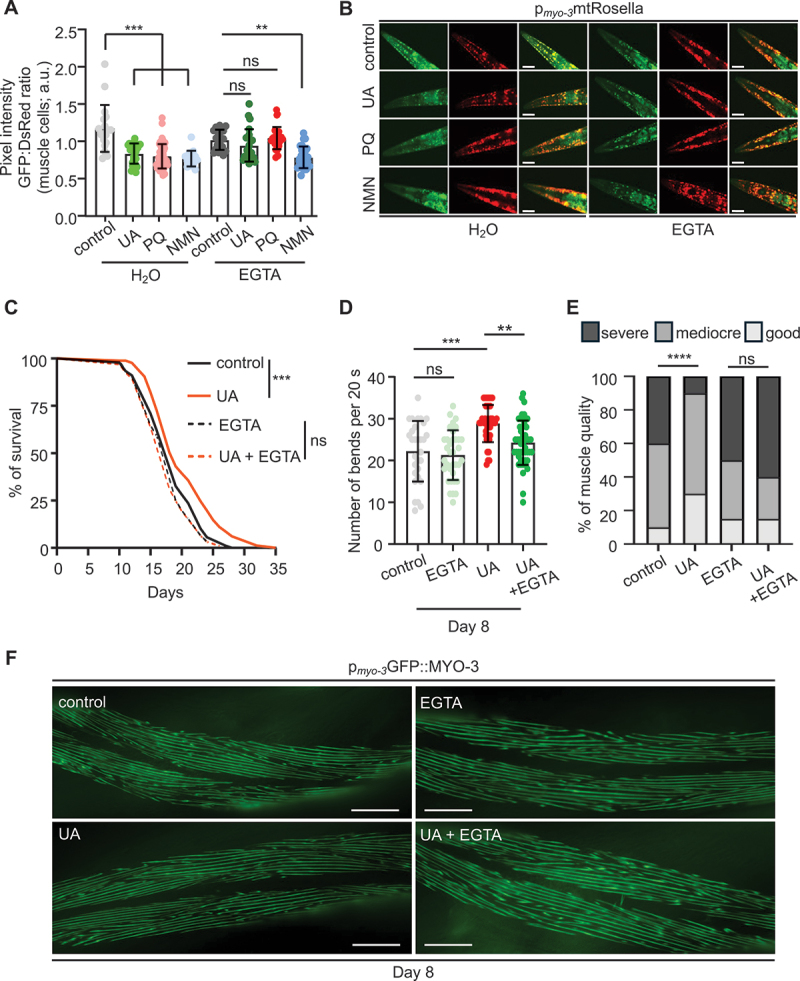
Urolithin A supplementation triggers calcium-mediated mitophagy to confer geroprotection. (A and B) Mitophagy levels estimated by the GFP:DsRed ratio (A) and representative images (B) of 4-day old transgenic nematodes expressing the double-fluorophore mtRosella in body-wall muscles (*myo-3* promoter), under control conditions or treated with UA, PQ, NMN in the presence or absence (H_2_O) of EGTA. Low ratios correspond to high levels of mitophagy (data presented as mean ± SD; ns *p* > 0.05, **p < 0.01, ***p < 0.001; one-way ANOVA). (C) Lifespan of wild-type (N2) nematodes under control conditions and treated with UA, in the presence or the absence of EGTA (ns *p* > 0.05, ***p < 0.001; log-rank (Mantel-Cox) test). (D) Motility of aged (8-day old) WT (N2) nematodes in a drop of isotonic liquid (M9), under control conditions and treated with UA, in the presence or the absence of EGTA (data presented as mean ± SD; ns *p* > 0.05, **p < 0.01, ***p < 0.001; one-way ANOVA). (E and F) Qualitative assessment (E) and representative images (F) of myofilaments in body wall muscles of aged (8-day old) nematodes, visualized by the expression of GFP::MYO-3/myosin, under control conditions and treated with UA, in the presence or the absence of EGTA (scale bars: 50 μm; ns *p* > 0.05, ****p < 0.0001; Chi-square test). Lifespan values are given in Table S2; assays were performed at 20°C.

We next examined whether increased calcium levels are necessary for mitophagy induction upon UA, PQ or NMN treatment. To assess mitophagy levels, we utilized transgenic nematodes expressing the mitophagy biosensor mtRosella in body-wall muscle cells and neurons [3,20,34,35]. Rosella is a fluorescent reporter that combines a rapidly maturing, pH-insensitive DsRed protein fused with a pH-sensitive GFP variant [35–37]; this configuration enables the assessment of mitophagy levels by measuring the ratio of GFP:DsRed fluorescence intensity. mtRosella-expressing nematodes were exposed to UA, PQ or NMN with or without EGTA treatment. Notably, calcium chelation abolished UA- and PQ-induced mitophagy, while it had no effect on NMN-induced mitophagy, underscoring that these molecules act through distinct pathways to trigger mitochondrial degradation (Figure 2A,B and Figure S2E-H). Next, we examined the impact of UA on mitochondrial number and activity. Consistent with previous studies [19], we found that mitochondrial content remained unchanged, whereas mitochondrial membrane potential was reduced in 1-day adult nematodes following UA treatment (Figure S1D and S2I, J). In contrast, UA supplementation prevented excessive mitochondrial accumulation and improved mitochondrial membrane potential in 8-day adult worms (Figure S1D and S2I, J). Interestingly, the effect of UA supplementation on mitochondrial activity was calcium-dependent, as co-treatment with EGTA suppressed this phenotype (Figure S2J). Taken together, these findings suggest that calcium signaling is required in UA-treated animals for mitophagy and mitochondrial homeostasis maintenance during ageing.

### Intracellular calcium elevation is essential for the healthspan and lifespan-extending properties of UA in *C. elegans*

Previous studies across a wide range of organisms, from nematodes to humans, have demonstrated that UA supplementation delays age-related muscle degeneration, extends healthspan and promotes longevity [19,25,26,38,39]. Given these findings, we sought to determine whether the elevation of calcium levels is a key mechanism underlying the beneficial effects of UA on organismal physiology.

To this end, we inhibited calcium elevation using the calcium chelator EGTA. Strikingly, EGTA administration significantly reduced the lifespan of UA-treated *C. elegans*, while it had no significant effect on the lifespan of untreated nematodes (Figure 2C). This finding suggests that the elevation of intracellular calcium is crucial for the longevity-promoting effects of UA. In line with previous studies showing that UA preserves muscle morphology and myofilament organization during ageing in *C. elegans* [19], we found that EGTA supplementation completely abrogated the positive effects of UA on the locomotion of aged nematodes (Figure 2D). Moreover, EGTA treatment disrupted myofilament organization in UA-treated worms (Figure 2E,F), further highlighting the role of calcium signaling in the maintenance of muscle integrity during ageing. Together, these results indicate that the elevation of intracellular calcium is essential not only for UA’s lifespan-extending properties in nematodes but also for preserving muscle function during ageing.

### UA triggers transcriptome and proteome adaptive responses to regulate calcium homeostasis and maintain organellar integrity and functional crosstalk

**Figure 3.**
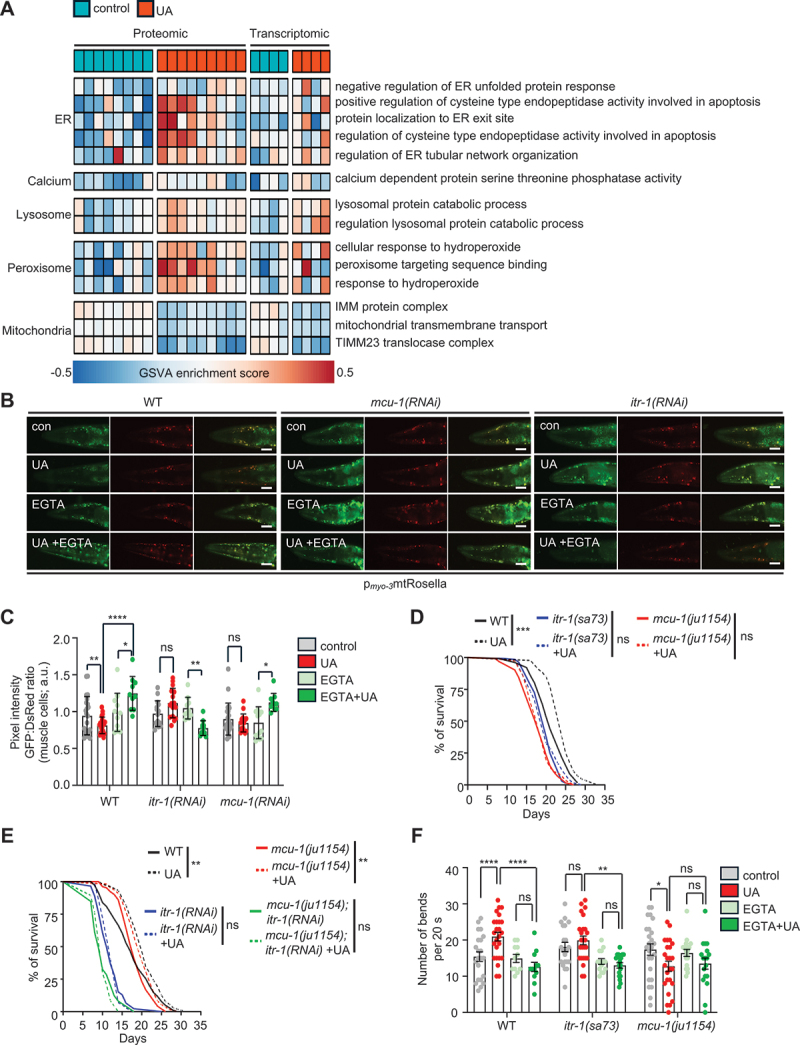
The ER-to-mitochondria calcium transport machinery is required for the beneficial effects of Urolithin A. (A) Heatmap of GSVA-enriched pathways related to ER, calcium, lysosomal, peroxisomal and mitochondrial functions based on proteomic and transcriptomic datasets obtained from UA-treated *versus* untreated N2 animals. Peptide sets within the proteomic dataset exhibiting *p* values < 0.05 were considered statistically significant and subsequently compared with those in the transcriptomic dataset. (B and C) Mitophagy levels estimated by the GFP:DsRed ratio (C) and representative images (B) of 4-day old transgenic nematodes expressing the double-fluorophore mtRosella in body-wall muscles (*myo-3* promoter), exposed to RNAi against *itr-1* or *mcu-1*, under control conditions and treated with UA, in the presence or absence of EGTA. Low ratios correspond to high levels of mitophagy (scale bars: 50 μm; data presented as mean ± SD; ns *p* > 0.05, *p < 0.05, **p < 0.01, ****p < 0.0001; two-way ANOVA). (D) Lifespan of WT (N2), *mcu-1(ju1154)* and *itr-1(sa73)* mutant nematodes under control conditions or treated with UA (ns *p* > 0.05, ***p < 0.001; log-rank (Mantel-Cox) test). (E) Lifespan of WT (N2) and *mcu-1(ju1154)* mutant nematodes exposed to RNAi against *itr-1*, under control conditions and treated with UA (ns *p* > 0.05, **p < 0.01; log-rank (Mantel-Cox) test). (F) Motility of aged (8-day old) WT (N2), *itr-1(sa73)* and *mcu-1(ju1154)* nematodes in a drop of isotonic liquid (M9), cultivated under control conditions or treated with UA, in the presence or the absence of EGTA (data presented as mean ± SD; ns *p* > 0.05, *p < 0.05, **p < 0.01, ****p < 0.0001; two-way ANOVA). Lifespan values are given in Table S2; assays were performed at 20°C.

To dissect the molecular responses underlying the UA-mediated beneficial effects on cellular function and organismal physiology, we conducted transcriptomic and proteomic analyses of wild-type nematodes treated with UA *versus* untreated controls. Principal-component analysis (PCA) of both transcriptomic and proteomic datasets revealed clear clustering between the control and UA-treated groups, indicating distinct molecular profiles in response to UA (Figure S1E, F). Transcriptomic analysis identified 936 differentially expressed genes (DEGs), including 660 upregulated and 276 downregulated genes (fold change, FC ≥ 1.2; *p* < 0.05; (Figure S1G)), while proteomic profiling identified 4603 nematode proteins, from which 809 were significantly upregulated and 510 downregulated (Figure S1H). Heatmaps of DEGs and differentially expressed peptides (DEPs) provide further insights into the impact of UA on gene and protein expression patterns (Figure S1I, J). To explore the functional significance of these adaptations, we performed Gene Ontology (GO) enrichment analysis including both upregulated and downregulated genes or proteins. The analyses revealed the enrichment of terms associated with key biological processes and cellular compartments, including calcium signaling, the ER, lysosomes, mitochondria and peroxisomes for DEGs and processes associated with calcium homeostasis, mitochondrial electron transport, protein processing in the ER, lysosomal activity and oxidative stress response for DEPs (Figure S1K, L). To better integrate the transcriptomic and proteomic analyses, we performed Gene Set Variation Analysis (GSVA) to assess pathway enrichment patterns in a sample-wise manner across both datasets [40]. We focused specifically on pathways relevant to the ER, calcium signaling, lysosomal function, peroxisomal dynamics, and mitochondrial activity. Only gene sets within the proteomic dataset exhibiting a *p* value < 0.05 were retained for cross-comparison. The resulting GSVA enrichment scores were visualized in a heatmap (Figure 3A), showing high concordance between transcriptomic and proteomic profiles across organelle-specific processes. These data provide evidence of coordinated transcriptome-proteome responses to UA, particularly in modules regulating organelle homeostasis and inter-organellar communication.

To further investigate the functional relationships among proteins altered by UA, we conducted a network analysis of the enriched proteomic data. Our analysis uncovered strong interconnectivity among mitochondrial, lysosomal and ER-associated pathways (Figure S1M). Indeed, several mitochondrial proteins associated with energy production (COX-5B, COX-4, COX-6A, COX-6B, COX-6C), metabolism (ACL-6, ACL-7, TRXR-1), and mitostasis (SLC-25A46) were significantly induced in UA-treated groups (Figure S3A). These findings could explain the increased mitochondrial membrane potential and improved mitochondrial content observed in UA-treated aged nematodes (Figure S1D and S2J). Additionally, our analysis suggests that UA supplementation modulates lysosomal function, as indicated by the elevated levels of several lysosomal proteins, including those with structural and enzymatic roles, such as DCT-11, LAAT-1, HRG-7, ASP-3 and ASP-12 (Figure S3B). Consistent with these results, we observed that UA improves lysosomal activity, by staining nematodes with a lysosome-specific dye (Figure S3C, D). Importantly, this enhancement of lysosomal function is also dependent on calcium elevation (Figure S3C, D).

Together, our multi-omics analysis provides a comprehensive overview of UA’s multifaceted impact on cellular metabolic pathways and organelles, particularly those involved in calcium homeostasis, such as the ER, mitochondria and lysosomes; further supporting our hypothesis that UA exerts its beneficial effects on cellular physiology through a broad proteostatic remodeling that sustains inter-organellar communication [41,42].

### ITR-1/ITPR/InsP3R and MCU-1/MCU regulate calcium signaling and lifespan upon UA treatment in *C. elegans*

Calcium signaling is a crucial regulator of multiple cellular processes, including energy metabolism, autophagy and cell death, all of which are tightly linked to cellular and organismal viability [14,29]. The regulation of intracellular calcium concentration relies on an intricate network of organelle-specific calcium channels and transporters. ER is the largest cellular Ca^2+^ storage site, capable of maintaining calcium homeostasis in cells [41,42]. Upon physiological stimuli, Ca^2+^ is rapidly released from the ER, initiating downstream signaling cascades that activate various calcium-dependent effector proteins such as kinases, phosphatases, and ion channels [14,29,41,42].

ITR-1/ITPR/InsP3R is a main regulator of calcium release from ER to cytosol [41–43]. We found that *itr-1*, the nematode homolog of the mammalian ITPR/InsP3R, was upregulated in our transcriptomic analysis, a finding further confirmed by increased mRNA levels through qRT-PCR validation (Figure S3E). Then, we asked whether ITR-1/ITPR/InsP3R mediates calcium elevation to promote mitophagy and longevity in response to UA supplementation. Notably, knocking down of *itr-1* suppressed both cytosolic calcium elevation and mitophagy induction in response to UA (Figure 3B, C and Figure S3F, G). Intriguingly, EGTA-mediated calcium chelation abolished UA-induced mitophagy in wild-type nematodes, but paradoxically increased mitophagy in ITR-1 deficient animals, possibly due to deregulated calcium distribution across organelles (Figure 3B,C).

Importantly, ITR-1 depletion abolished UA-mediated improvements in myofilament structure and lifespan extension, underscoring that ITR-1 is indispensable for the geroprotective effects of UA (Figure 3D,E and Figure S3H, I). While proteomic analysis did not show any significant changes in ITR-1/ITPR/InsP3R protein expression levels post-UA treatment, it revealed elevated levels of ER proteins involved in lipid metabolism (FAT-5, ACS-22), proteostasis (SRP-1, ERO-1, DNJ-2, XBP-1) and ER morphology and organization (TMCO-1/F22B5.10, EMC-3, ERL-1) (Figure S3J) [44–49]. Recent studies suggest that both the EMC complex and TMCO1 are essential for calcium release from the ER to the cytosol [44,49]. Although UA did not alter the mRNA levels of both *emc-3* and *tmco-1/F22B5.10* (the nematode homologs of the mammalian *EMC3* and *TMCO1* respectively), its supplementation failed to extend lifespan and preserve muscle function during ageing in nematodes depleted of TMCO-1/F22B5.10 and EMC-3 (Figure S3K-M). These findings indicate that calcium release from the ER is a critical early step in promoting healthspan and longevity in response to UA treatment.

Calcium transfer from the ER to mitochondria occurs at MAMs, where ITR-1/ITPR/InsP3R aligns with VDACs (voltage dependent anion channels), which mediate the fast transfer of Ca^2+^ across the outer mitochondrial membrane (OMM), followed by its accumulation in the mitochondrial matrix via the MCU (mitochondrial calcium uniporter) of the inner mitochondrial membrane (IMM) [41,42,50]. Consistently, UA increased ER-mitochondria contact sites and mitochondrial calcium uptake (Figure 1A,B,E,F), prevented age-related structural decline in organelles, restored mitochondrial network, ER sheet integrity, and lysosomal morphology (Figure S1A-C).

To assess whether mitochondrial calcium uptake is also essential for mitophagy induction and lifespan extension upon UA, we knocked down *mcu-1*, the nematode homolog of the mammalian MCU. Similarly to ITR-1, MCU-1 depletion abolished UA-mediated mitophagy, while it did not display any additive effect in combination with calcium chelation, suggesting that MCU-1/MCU and Ca^2+^ act in the same pathway to mediate the effect of UA (Figure 3B,C). Notably, genetic ablation of *mcu-1* inhibited UA-mediated longevity (Figure 3D,E). Furthermore, simultaneous depletion of *mcu-1* and *itr-1* did not produce an additive effect, suggesting that both ITR-1 and MCU-1 act within the same pathway to promote lifespan extension upon UA treatment (Figure 3E). Intriguingly, UA retained some of its beneficial effect in *mcu-1(ju1154)* animals when they were fed with HT115 bacteria for RNAi, suggesting that differences in the metabolic profiles between food sources might activate alternative pathways for mitochondrial calcium import [50–52]. Beyond longevity, both ITR-1 and MCU-1 are also required for UA-induced improvements in healthspan, as UA failed to restore age-related locomotion defects in *mcu-1(ju1154)* and *itr-1(sa73)* mutant nematodes (Figure 3F). Additionally, calcium chelation did not further diminish locomotion in the UA-treated *mcu-1(ju1154)* and *itr-1(sa73)* mutants, suggesting that calcium release from the ER via ITR-1/ITPR/InsP3R and subsequent mitochondrial uptake through MCU-1/MCU are jointly essential for the UA-mediated healthspan and lifespan extension (Figure 3E,F). Interestingly, MCU-1 depletion suppressed the UA-induced increase in cytosolic calcium, indicating that mitochondrial calcium cycling could contribute to cytosolic calcium elevation, which, in turn, could trigger mitophagy (Figure S3F, G). These results highlight a coordinated mechanism in which calcium release from the ER via ITR-1/ITPR/InsP3R and mitochondrial uptake via MCU-1/MCU are essential for the beneficial effects of UA on mitophagy, healthspan and lifespan in *C. elegans*.

### DRP-1/DNM1L/DRP1-mediated mitophagy promotes lifespan extension and sustains muscle function upon UA treatment in *C. elegans*

Mitochondrial quality control mechanisms are essential for the maintenance of cellular health and tissue integrity [16, 53–55]. Mitochondrial dynamics, comprising fission and fusion processes, enable the constant remodeling of the mitochondrial network to adapt to metabolic cellular demands and remove damaged components [55,56]. Particularly, mitochondrial fission is a critical prerequisite for mitophagy, as it divides the network into smaller fragments that can be selectively degraded [57,58]. It is suggested that smaller mitochondria can be easily engulfed by autophagosomes to be eventually degraded and recycled [57, 59–61]. Mitochondrial fission largely depends on DNM1L/DRP1, which constricts and severs mitochondrial membranes, thereby priming the organelle for degradation [62,63]. However, mitophagy can proceed through both DNM1L/DRP1-dependent and -independent pathways, underlining the existence of alternative mechanisms to promote mitophagy and sustain energy metabolism [16,64].

**Figure 4.**
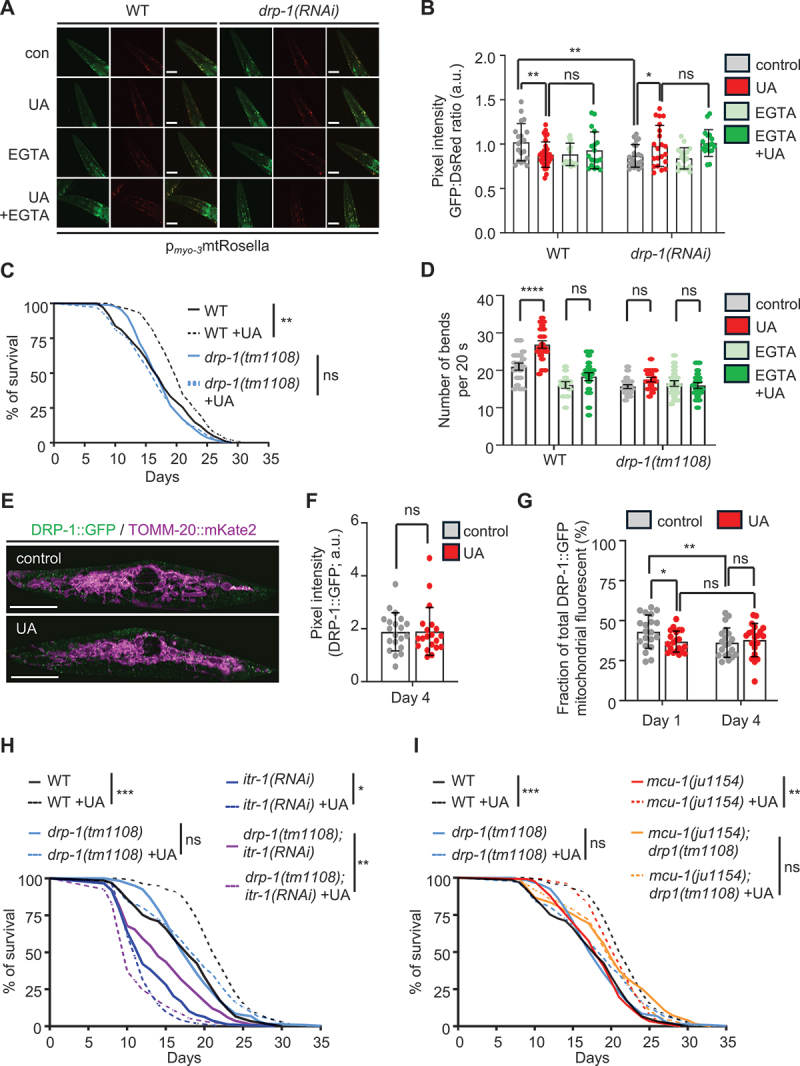
DRP-1 is required for UA-mediated mitophagy, lifespan extension and muscle function maintenance during ageing. (A and B) Representative images (A) and mitophagy levels estimated by the GFP:DsRed ratio (B) of 4-day old transgenic nematodes expressing the double-fluorophore mtRosella in body-wall muscles (*myo-3* promoter), treated with RNAi against *drp-1*, under control conditions or supplemented with UA. Scale bars: 50 μm (data presented as mean ± SD; ns *p* > 0.05, *p < 0.05, **p < 0.01; two-way ANOVA). (C) Lifespan assay of WT (N2) and *drp-1(tm1108)* mutant nematodes under control conditions or treated with UA (ns *p* > 0.05, **p < 0.01; log-rank (Mantel-Cox) test). (D) motility assessment of 8-day old WT (N2) and *drp-1(tm1108)* nematodes under control conditions or treated with UA, in the presence or absence of EGTA (data presented as mean ± SD; ns *p* > 0.05, ****p < 0.0001; two-way ANOVA). (E-G) representative merged images of 1-day old animals (E) and quantification of total DRP-1 levels (data presented as mean ± SD; ns *p* > 0.05, unpaired *t-test*) (F) as well as of the percentage of mitochondria-localized DRP-1 (data presented as mean ± SD; ns *p* > 0.05, *p < 0.05, **p < 0.01; two-way ANOVA) (G) in transgenic nematodes co-expressing a DRP-1::GFP translational fusion from the endogenous locus (green) and a mitochondria-targeted mKate2 (magenta) in body wall muscles (*myo-3* promoter) under control conditions or supplemented with UA. Scale bars: 40 μm. (H and I) Lifespan curves of WT (N2) and *drp-1(tm1108)* mutant nematodes treated with RNAi against *itr-1* (H) and *mcu-1(ju1154)*, *drp-1(tm1108)*, and *mcu-1(ju1154);drp-1(tm1108)* mutants (I) under control conditions or following supplementation with UA (significance is presented in comparison to the respective untreated control; ns *p* > 0.05, *p < 0.05, **p < 0.01, ***p < 0.001; log-rank (Mantel-Cox) test).

Therefore, we asked whether DRP-1 is required for UA-induced mitophagy in *C. elegans*. Our results showed that DRP-1/DNM1L/DRP1 deficiency completely abolished mitophagy induction in both body-wall muscle and neuronal cells in response to UA (Figure 4A, B and Figure S4A, B). Notably, EGTA treatment had no additional impact in the absence of DRP-1, suggesting that calcium elevation and DRP-1-dependent fission converge on the same pathway. Further investigating the interplay between DRP-1 function and calcium homeostasis, we monitored cytosolic Ca^2+^ levels under conditions of DRP-1 depletion. Interestingly, knocking down of *drp-1* alone increased the baseline calcium levels and eliminated the cytosolic calcium elevation typically observed upon UA treatment, implying that DRP-1 may also influence baseline calcium homeostasis, potentially as an upstream modulator (Figure S4C, D). We next evaluated mitochondrial network morphology in body-wall muscle cells at day 4 of adulthood, a timepoint when structural deterioration typically becomes apparent. UA treatment reduced age-related fragmentation of the mitochondrial network in a calcium-dependent manner (Figure S4E, F). In contrast, DRP-1 depletion led to elongated and interconnected mitochondria unresponsive to UA or EGTA. Only a mild increase in fragmentation was observed with both treatments combined, reinforcing the notion that DRP-1 function is essential for UA-driven mitochondrial remodeling (Figure S4E, F).

Lifespan values are given in Table S1; assays were performed at 20°C.

We also investigated the implication of DRP-1/DNM1L/DRP1 in UA-induced lifespan extension. Loss of DRP-1/DNM1L/DRP1 inhibited the lifespan-extending effects of UA (Figure 4C). Additionally, we assessed the involvement of DRP-1/DNM1L/DRP1 in UA’s geroprotective effects by analyzing locomotion and myofilament morphology. We observed that in the absence of DRP-1, neither locomotion nor myofilament structure were restored upon UA treatment (Figure 4D, Figure S4G, H). Furthermore, EGTA treatment did not further impair mobility in DRP-1/DNM1L/DRP1-deficient nematodes, suggesting that DRP-1/DNM1L/DRP1 activity is stimulated downstream to UA-mediated calcium elevation (Figure 4D).

Recent studies suggest that DNM1L/DRP1 abundance, localization and activity are modulated by post-translational modifications [65,66]. To examine the potential regulatory modifications in DRP-1, we performed mass spectrometry analysis on immunoprecipitated DRP-1::GFP from untreated and UA-treated nematodes. No differential phosphorylation or other post-translational modifications were detected. Moreover, the total DRP-1::GFP levels and their mitochondrial localization remained unchanged with UA, as confirmed by immunoblotting (Figure S4I-K). Consistently, we did not detect any difference either in the total fluorescence of DRP-1::GFP reporter or in the fraction that colocalizes with mitochondria in muscle cells of animals treated with UA, compared to their respective controls (Figure 4E–G, Figure S4L).

To dissect the genetic pathway underlying UA’s geroprotective effects, we then performed epistatic analyses of DRP-1/DNM1L/DRP1 with ITR-1/ITPR/InsP3R and/or MCU-1/MCU. Lifespan assays revealed that simultaneous loss of either ITR-1 or MCU-1 alongside DRP-1 abolished the UA lifespan-extending effect without any additive interaction, indicating that DRP-1/DRP1, ITR-1/ITPR/InsP3R, and MCU-1/MCU act together to mediate longevity in response to UA supplementation (Figure 4H, I). Interestingly, we found that UA treatment shortened the lifespan of *drp-1(tm1108);itr-1(RNAi)* nematodes (Figure 3H), suggesting that the absence of both ITR-1 and DRP-1 leads to uncontrolled disruption of mitochondrial dynamics and/or calcium homeostasis; under these conditions UA supplementation results in detrimental outcomes for lifespan.

Together, these findings highlight that DRP-1 is indispensable for UA-mediated mitophagy, mitochondrial remodeling, and the maintenance of muscle function and lifespan extension. Although UA does not directly modulate DRP-1 levels or localization, its effects require a functional baseline of DRP-1 activity, placing DRP-1 as a central effector in the calcium-dependent pathway through which UA promotes healthy ageing.

### UA induces SKN-1 activity in a calcium-dependent manner to promote mitochondrial biogenesis

Organismal homeostasis relies heavily on the intricate coordination between mitophagy and mitochondrial biogenesis to sustain energy metabolism [3,67,68]. These two interconnected processes ensure that damaged mitochondria are effectively removed, while new functional mitochondria are generated to maintain energy metabolism and cellular integrity. Notably, several studies in *Drosophila melanogaster* as well as in mammals underscored the key role of NFE2L2/Nrf2 in promoting mitochondrial biogenesis by transcriptionally regulating various nuclear-encoded genes involved in mitochondrial function [68–75]. SKN-1, the nematode homolog of NFE2L2/Nrf2, has been found to associate with OMM proteins and regulate mitochondrial function, oxidative stress responses and longevity [76–78]. Moreover, SKN-1 is shown to coordinate mitophagy and mitochondrial biogenesis via the transcriptional regulation of the mitophagy receptor protein DCT-1/BNIP3 [3].

**Figure 5.**
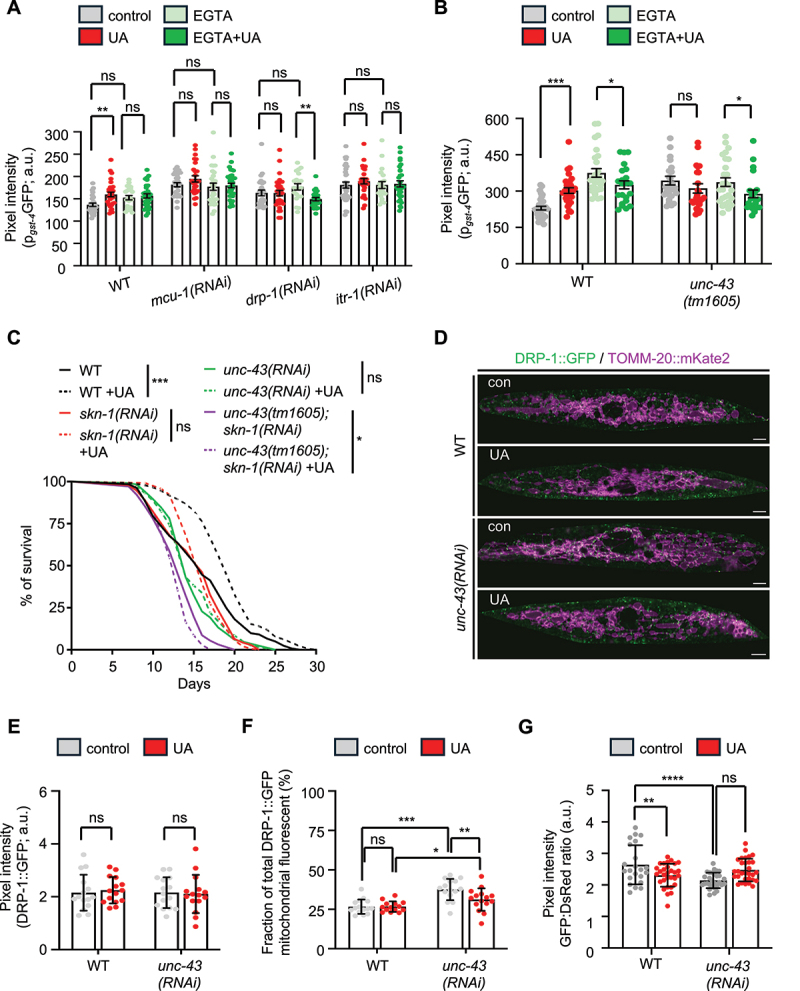
SKN-1 is activated upon UA supplementation and is required for mitophagy induction and lifespan extension. (A and B) Fluorescence intensity of transgenic nematodes expressing the p_*gst-4*_GFP transgene reporter upon RNAi-mediated knockdown of *mcu-1*, *itr-1* or *drp-1*, under control conditions or supplemented with UA, in the presence or absence of EGTA (A) as well as in *unc-43(tm1605)* mutants under the same conditions (B) (data presented as mean ± SD; ns *p* > 0.05, *p < 0.05, **p < 0.01, ***p < 0.001; two-way ANOVA). (C) Lifespan curves of WT (N2) and *unc-43(tm1605)* mutant nematodes treated with RNAi against *skn-1* under control conditions or supplemented with UA (significance is presented in comparison to the respective untreated control; ns *p* > 0.05, *p < 0.05, ***p < 0.001; log-rank (Mantel-Cox) test). (D-F) Merge of representative images (scale bars: 5 μm) (D) and quantification of total DRP-1 levels (data presented as mean ± SD; ns *p* > 0.05, unpaired *t-test*) (E) or of the percentage of mitochondria-localized DRP-1 (data presented as mean ± SD; ns *p* > 0.05, *p < 0.05, **p < 0.01, ***p < 0.001; two-way ANOVA) (F) in 1-day old transgenic nematodes co-expressing a DRP-1::GFP translational fusion from the endogenous locus (green) and a mitochondria-targeted mKate2 (magenta) in body wall muscles (*myo-3* promoter), subjected to RNAi against *unc-43* under control conditions or supplemented with UA . (G) Mitophagy levels estimated by the GFP:DsRed ratio of 4-day old transgenic nematodes expressing the double-fluorophore mtRosella in body-wall muscles (*myo-3* promoter), exposed to RNAi against *unc-43*, under control conditions and treated with UA. Low ratios correspond to high levels of mitophagy (data presented as mean ± SD; ns *p* > 0.05, **p < 0.01, ****p < 0.0001; two-way ANOVA). Lifespan values are given in Table S1; assays were performed at 20°C.

SKN-1 was also found to be necessary for the UA-induced lifespan extension in *C. elegans* (Figure S5A), as previously reported [19]. Moreover, UA supplementation induced SKN-1 activation but did not affect DAF-16 stimulation and HLH-30 nuclear translocation, the nematode homologs of FOXO and TFEB, respectively (Figure 5A, Figure S3N-P and Figure S5B). Notably, NMN supplementation did not impact the transcriptional activation of SKN-1 or DAF-16, or HLH-30 nuclear translocation, further underscoring NMN’s distinct mechanism of action as compared to UA (Figure S3N-P and Figure S5C). Although increased ROS levels are known to promote SKN-1 induction, we found that NAC supplementation did not abolish UA-mediated SKN-1 activity, suggesting that ROS signaling is not involved in this process (Figure S5B). This notion is supported by evidence that ellagitannins, ellagic acid, and their metabolites, such as urolithins, exhibit antioxidant properties [79, 80], suggesting the presence of an alternative signaling mediator.

To further explore the mechanism underlying SKN-1 activation in response to UA, we investigated the role of calcium signaling. EGTA supplementation effectively blocked SKN-1 activation following UA treatment (Figure 5A, Figure S3N and Figure S5C), suggesting that calcium signaling plays a crucial role in UA-induced SKN-1 activation. Furthermore, the simultaneous depletion of ITR-1/ITPR/InsP3R, MCU-1/MCU or DRP-1/DNM1L/DRP1 along with calcium chelation did not lead to any additional inhibition of SKN-1 activity upon UA treatment (Figure 5A). These findings indicate that UA induces calcium release from the ER to mitochondria, thereby facilitating mitophagy and mitochondrial biogenesis through SKN-1 activation.

In mammals, cytoplasmic calcium elevation influences mitochondrial dynamics and stimulates mitochondrial biogenesis through the activation of CAMK2 (calcium/calmodulin dependent protein kinase II), MAPK/p38 (mitogen-activated protein kinase) and PPARGC1A/PGC1α (PPARG coactivator 1 alpha) [81,82]. UNC-43, the homolog of CAMK2D, regulates SKN-1 activity to promote mitochondrial biogenesis, highlighting the crosstalk between calcium signaling and SKN-1 induction [3]. Thus, we asked whether UNC-43/CAMK2D is required for SKN-1 activation upon UA administration. We found that UNC-43 deficiency abolished UA-mediated SKN-1 activity (Figure 5B). Furthermore, UNC-43 acts in parallel with calcium elevation to trigger SKN-1, as simultaneous calcium chelation and UNC-43 depletion did not result in additional effect on SKN-1 (Figure 5B). Lifespan assays further revealed that UNC-43 and SKN-1 function together to mediate the longevity effects induced by UA supplementation (Figure 5C).

Given the known functional interplay between CAMK2, mitochondrial dynamics, and mitophagy in response to diverse stimuli [82–88], we investigated the relationship between UNC-43/CAMK2D and DRP-1/DNM1L/DRP1 upon UA treatment. A previous study in nematodes has shown that UNC-43 phosphorylates and negatively regulates DRP-1 activity to prevent excessive mitochondrial fragmentation under normal conditions [65][]. Although we did not detect changes in DRP-1 phosphorylation status upon UA treatment, we observed that depletion of UNC-43 significantly increased mitochondrial DRP-1::GFP localization both under basal and UA-treated conditions (Figure 5D–F and Figure S5D). This finding indicates that UNC-43 exerts inhibitory control over DRP-1 localization under normal conditions, likely regulating basal mitophagy levels. Indeed, we found that UNC-43 deficiency led to elevated mitochondrial degradation in both muscle and neuronal cells and this effect was not further increased by UA treatment, consistent with a model where UNC-43 acts as a negative regulator of basal DRP-1-dependent mitophagy (Figure 5G and Figure S5E-G). Further investigation of the UNC-43/CAMK2D impact on UA-improved healthspan revealed that UNC-43 was not required for the maintenance of myofilament structure during ageing, while age-related decline in motility was not improved in UNC-43-depleted nematodes upon UA treatment (Figure S5H-J). These findings suggest that the role of UNC-43 is specifically tied to mitochondrial biogenesis rather than structural maintenance in response to UA supplementation.

Overall, these findings provide significant insights into the highly coordinated complex mechanisms driving UA-mediated activation of SKN-1/NFE2L2/Nrf2 and its pivotal role in the maintenance of mitochondrial homeostasis and organismal health. Notably, our results reveal that UA activates SKN-1 independently of ROS signaling, highlighting a previously unrecognized, calcium-dependent pathway involving UNC-43/CAMK2D. This pathway not only drives mitophagy but also enhances mitochondrial biogenesis, underscoring the dual role of SKN-1 in both mitochondrial quality control and renewal [3,67]. The involvement of calcium signaling and UNC-43/CAMK2D in SKN-1/NFE2L2/Nrf2-mediated processes emphasize the need for finely tuned regulatory networks, where calcium dynamics act as a key modulator to integrate various cellular stress responses, ensuring the coordination between mitochondrial degradation and biogenesis. This balanced regulation is essential for energy homeostasis, particularly during ageing, where mitochondrial functionality is compromised.

### UA triggers mitophagy and prevents stress-induced premature senescence in a calcium-dependent manner in normal human cells

To investigate whether the beneficial effects of UA on inter-organellar communication observed in *C. elegans* are conserved in mammals, we re-analyzed publicly available gene expression profiles from HT29 (human colorectal adenocarcinoma cell line with epithelial morphology) cells treated with vehicle or UA (50 μM), generated in a recent study evaluating gut barrier integrity upon UA supplementation [22]. PCA revealed a clear separation between control and UA-treated samples, indicating substantial transcriptional reprogramming (Figure S6A). Differential gene expression analysis identified 187 significantly upregulated genes and 50 downregulated genes (*p* < 0.05; fold change (FC) ≥ 1.2) in UA-treated cells (Figure S6B). GO enrichment analysis revealed that differentially expressed genes were significantly associated with key biological pathways, including calcium signaling, ER function, lysosomal activity, mitochondrial homeostasis, and peroxisomal function (Figure S6C). Notably, pathways related to ER stress responses, calcium-dependent protein binding, lysosomal membrane integrity, and mitochondrial membrane potential were prominently enriched, supporting the idea that UA modulates inter-organellar crosstalk in a conserved manner across species.

**Figure 6.**
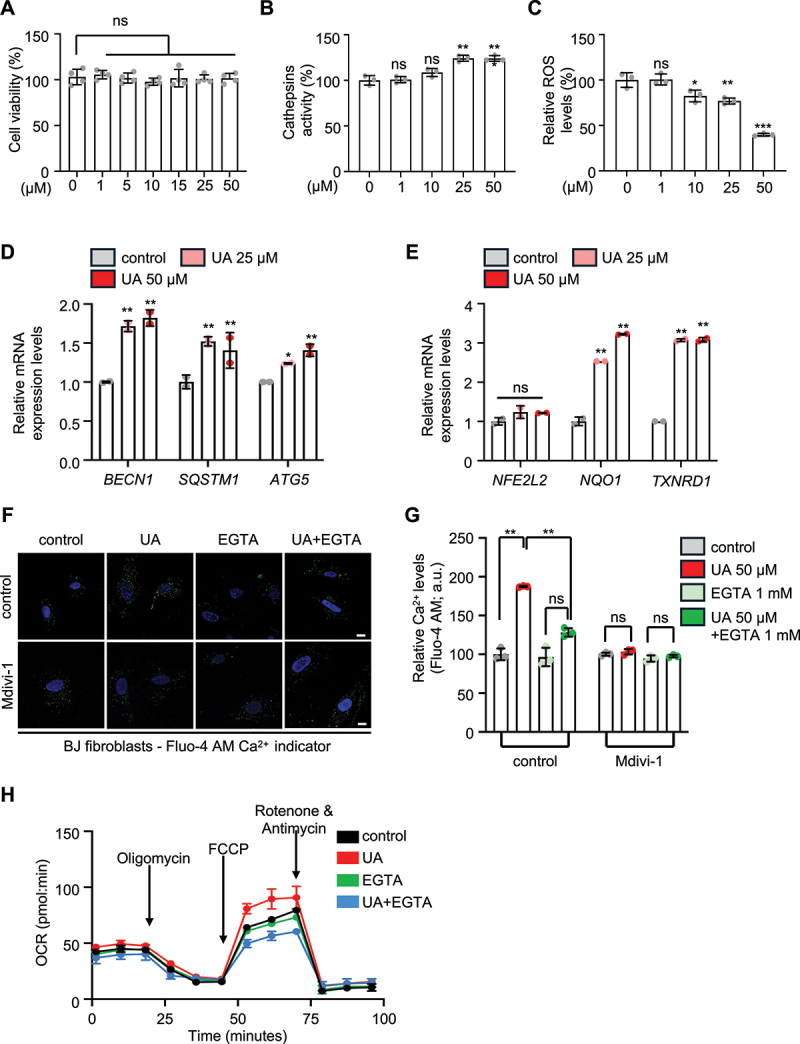
Urolithin A modulates mitochondrial homeostasis and calcium levels in normal human diploid skin fibroblasts (BJ cells). (A) Cell viability upon UA treatment at a wide range of different concentrations (data presented as mean ± SD; ns *p* > 0.05; one-way ANOVA). (B) Enzymatic activity of cathepsins upon supplementation with a range of UA concentrations (data presented as mean ± SD; ns *p* > 0.05, **p < 0.01; one-way ANOVA). (C) Endogenous oxidative load (ROS levels) upon supplementation with a range of UA concentrations (data presented as mean ± SD; ns *p* > 0.05, *p < 0.05, **p < 0.01, ***p < 0.001; one-way ANOVA). (D and E) relative mRNA levels of autophagy- (D) and oxidative response- (E) related genes upon UA administration (data presented as mean ± SEM; ns *p* > 0.05, * *p* < 0.05, ** *p* < 0.01, one-way ANOVA). (F and G) CLSM representative images (F) and quantification (G) of calcium levels estimated by Fluo-4 AM staining (green), under control conditions or following treatment with UA, in the presence or the absence of EGTA and supplementation with the DRP-1 inhibitor Mdivi-1. Nuclei were counterstained with DAPI (blue) (scale bars: 10 μm; data presented as mean ± SD; ns *p* > 0.05, ** *p* < 0.01, one-way ANOVA). (H) Oxygen consumption rates (seahorse analyzer) under control conditions and treated with UA, in the presence or the absence of EGTA (mean of OCR ± SD).

Given the essential role of calcium signaling in mediating the beneficial effects of UA in *C. elegans*, we assessed whether similar calcium-dependent mechanisms operate in mammalian cells. To mimic physiological conditions as closely as possible, we employed normal human cell lines, including human diploid fibroblasts (BJ cells) or endothelial cells (HUVEC). UA treatment across a broad concentration range (0–50 μM) did not impair viability in either cell type (Figure 6A and Figure S6D), indicating that UA is tolerated at these doses. Prompted by lysosome-related GO enrichment results, we examined UA effects on lysosomal function in BJ and HUVEC cells (Figure 3A and Figure S3B-D, S6C). Our results revealed that UA enhanced the enzymatic activity of cathepsins in BJ fibroblasts and HUVEC (Figure 6B and Figure S6E), aligning with a recent study which showed that UA supplementation improved cognitive functions and olfactory ability in an Alzheimer disease (AD) mouse model by restoring lysosomal function, particularly through the activity of CTSZ (cathepsin Z) [24]. Additionally, UA supplementation suppressed cell oxidative load and upregulated dose-dependently the expression of several oxidative stress response and autophagy-related genes in both fibroblasts and endothelial cells (Figure 6C–E and Figure S6F-H). Consistently, immunoblotting further confirmed autophagy induction via increased LC3B-II levels in both cell types (Figure S6I).

We next investigated whether UA induces calcium signaling in human cells. Indeed, UA treatment increased cytosolic calcium levels in multiple mammalian cell types, including BJ fibroblasts, HUVEC, HMC3 human microglial cells and mouse embryonic fibroblasts (MEFs) (Figure 6F, G and Figure S6J-N), consistent with the results in *C. elegans*. While UA did not significantly affect total mitochondrial pool in BJ fibroblasts and HUVEC, it enhanced mitochondrial respiration upon stress, an effect that was abolished by calcium chelation (Figure 6H and Figure S6O, P). Additionally, TMRE staining demonstrated that UA improved mitochondrial membrane potential without increasing mitochondrial ROS, confirming the calcium-dependent improvement in mitochondrial function (Figure S6Q-S).

**Figure 7.**
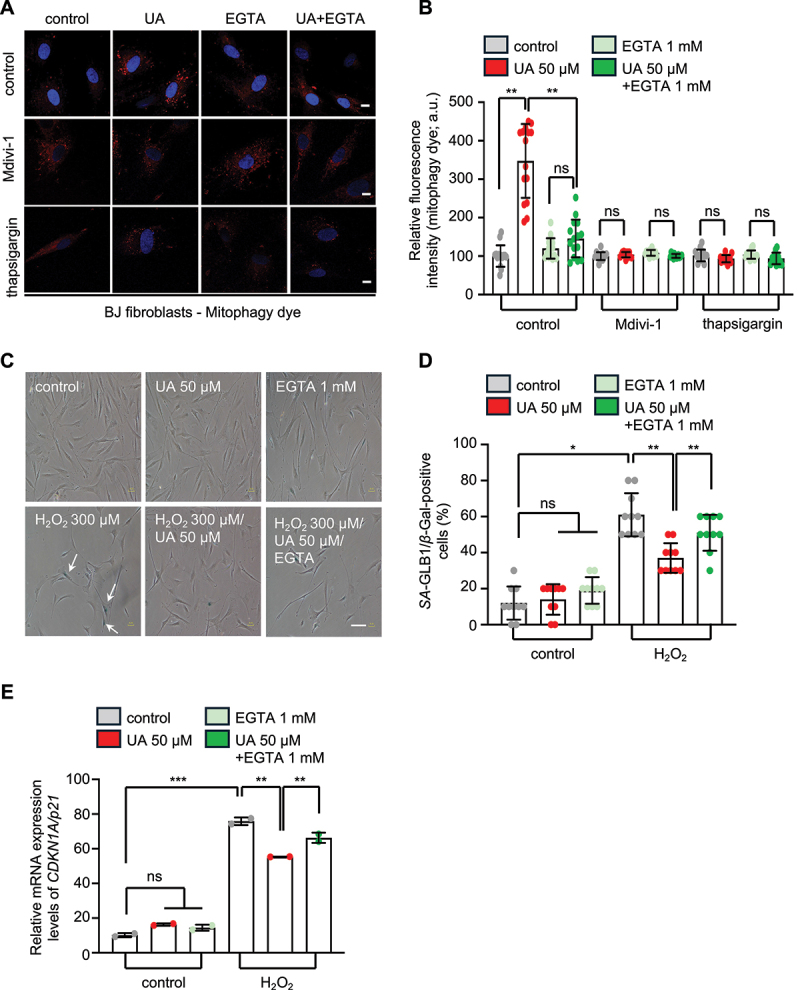
Urolithin A triggers mitophagy in normal human diploid skin fibroblasts (BJ cells) and prevents stress-induced premature senescence in a calcium dependent manner. (A and B) CLSM representative images (A) and quantification of mitophagy levels (B) as evident by fluorescence intensity of the Dojindo mitophagy dye (red), under control conditions or upon UA treatment , in the presence or the absence of EGTA and supplementation with the DRP-1 inhibitor Mdivi-1 or the SERCA inhibitor thapsigargin. Nuclei were counterstained with DAPI (blue) (scale bars: 10 μm; data presented as mean ± SD; ns *p* > 0.05, **p < 0.01, two-way ANOVA). (C and D) Relative number of *SA*-GLB1/*β*-gal-positive cells (mean of 10 optical fields) (C) and representative light field images (D) of cells under control conditions or upon UA treatment , in the presence or the absence of EGTA, with and without H_2_O_2_ (three exposures of 48 h each) (scale bars: 50 μm; data presented as mean ± SD; ns *p* > 0.05, *p < 0.05, **p < 0.01, one-way ANOVA). (E) Relative mRNA levels of *CDKN1A/p21* under control conditions or after treatment with UA, in the presence or the absence of EGTA, with and without H_2_O_2_ (used to induce SIPS; data presented as mean ± SEM; ns *p* > 0.05, **p < 0.01, ***p < 0.001; two-way ANOVA).

To test whether calcium signaling is required for UA-induced mitophagy, we used a mitophagy-specific dye that fluoresces upon lysosomal fusion with mitochondria [89, 90]. UA significantly increased mitophagic flux and lysosomal activity in both BJ fibroblasts and HUVEC, an effect that was abrogated by EGTA treatment, confirming the dependency on calcium (Figure 7A, B and Figure S6T-V). To dissect the mechanism behind UA-dependent mitophagy induction, we examined the involvement of ER-mitochondria calcium dynamics. Interestingly, treatment with thapsigargin, an ATP2A/SERCA inhibitor that depletes ER calcium stores, inhibited UA-induced mitophagy (Figure 7A,B and Figure S6U, V) [91]. This finding suggests that targeted calcium transfer from ER to mitochondria, rather than generalized cytosolic calcium elevation is necessary for UA-mediated mitophagy activation. These findings align with our *C. elegans* data showing that UA enhances MAMs, providing structural support for targeted calcium signaling (Figure 1A,B).

Moreover, we assessed the role of DNM1L/DRP1-mediated mitochondrial fission in UA-induced mitophagy. Pharmacological inhibition of DNM1L/DRP1 using Mdivi-1 blocked mitophagy in BJ and HUVEC cells and abolished UA-induced calcium elevation (Figures 6F,G, 7A,B and Figure S6J, K, U, V) [92–94]. These effects phenocopy our observations in *C. elegans*, where DRP-1 knockdown impaired calcium elevation and mitophagy, reinforcing DNM1L/DRP1 as a central regulator in this calcium-dependent pathway (Figure 4A, B and Figure S4A-D).

Given that mitochondrial dysfunction is a well-known ageing hallmark, we then asked whether UA-mediated mitophagy activation would affect senescence. Thus, we assessed the potential role of UA in preventing Stress-Induced Premature Senescence (SIPS). Consistent with recent studies showing that UA exerts senolytic properties [38, 95–97], we found that UA treatment reduced the proportion of SA-GLB1/β-Gal-positive cells in BJ fibroblasts exposed to hydrogen peroxide (H_2_O_2_) in a calcium-dependent manner (Figure 7C, D). Under these conditions, UA supplementation also partially decreased the upregulated *CDKN1A/p21* mRNA expression (a well-established marker of cellular senescence); this decrease was reversed upon calcium chelation in both cell types (Figure 7E and Figure S6W), suggesting that the transcriptional modulation of senescence markers by UA is also dependent on calcium elevation.

Collectively, these results demonstrate that UA exerts potent, calcium-dependent effects on mitophagy, mitochondrial function, and cellular resilience across species. By promoting inter-organellar calcium transfer and engaging mitochondrial dynamics, UA triggers quality control programs that enhance mitochondrial health and suppress premature senescence. These conserved mechanisms highlight UA’s therapeutic potential in mitigating the age-related decline of cellular functionality supporting the broader concept that calcium signaling serves as a central integrator of mitochondrial homeostasis and longevity pathways.

## Discussion

Ageing is characterized by a systemic deterioration of cellular homeostasis and tissue integrity, largely driven by mitochondrial dysfunction and impaired inter-organellar communication. A growing body of evidence suggests that the dynamic physical and functional interplay between mitochondria, ER and lysosomes is critical for the maintenance of energy metabolism, proteostasis and stress resilience [1, 2, 7, 8, 11–13, 42, 98]. Disruption of these intricate inter-organellar networks is increasingly recognized as a fundamental mediator of age-related decline [7, 11–13]. However, the molecular mechanisms underlying the maintenance and restoration of inter-organellar communication still remain elusive.

Our study uncovers the central role of mitophagy as a critical denominator of inter-organellar crosstalk and rejuvenation. UA has emerged as a potent modulator of mitophagy, enhancing mitochondrial quality and overall healthspan across species from *C. elegans* to humans [19, 20, 28]. We demonstrate that UA restores ER-mitochondria-lysosome crosstalk during ageing through a calcium-dependent signaling pathway. Importantly, we reveal that mitophagy not only selectively eliminates damaged mitochondria but also remodels organellar architecture, facilitating the reestablishment of functional contact sites between intracellular compartments. These findings position UA as a potent regulator of organellar homeostasis and provide new insights into how targeted mitophagy induction can promote healthy ageing.

**Figure 8.**
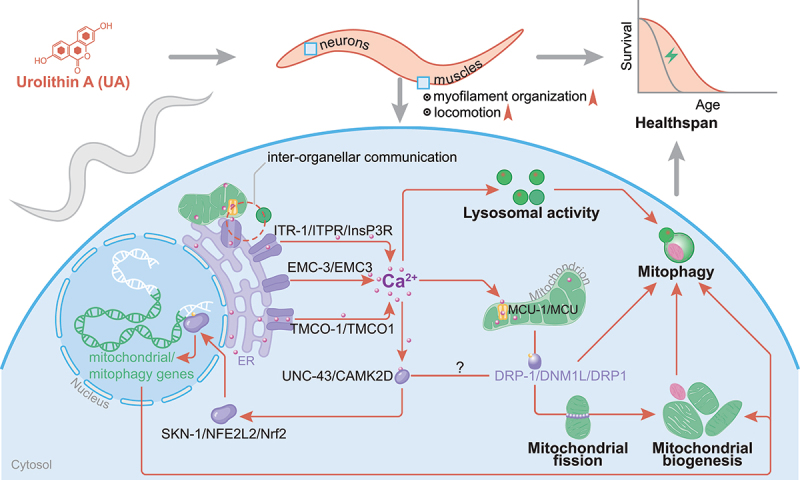
Urolithin A rewires inter-organellar communication to promote longevity. UA promotes mitophagy and longevity through calcium-dependent inter-organellar communication mechanisms. Upon UA supplementation, calcium is released from the endoplasmic reticulum (ER) into the cytosol via channels, such as ITR-1/ITPR/InsP3R, EMC-3/EMC3, and TMCO-1/TMCO1. Subsequent elevation of cytosolic calcium enhances lysosomal activity, which is essential for initiating mitophagy. Calcium is also taken up by mitochondria through the mitochondrial calcium uniporter MCU-1/MCU, thereby facilitating DRP-1/DNM1L/DRP1-mediated mitochondrial fission. Mitochondrial fission is a critical step for mitophagy, mediating the isolation of damaged organelles and their subsequent degradation. In addition to promoting mitophagy, calcium elevation triggers mitochondrial biogenesis via activation of UNC-43/CAMK2D and SKN-1/NFE2L2/Nrf2, thereby replenishing the mitochondrial pool and supporting cellular energy demands. The transcriptional activation of mitochondrial and mitophagy-related genes sustains cellular homeostasis and promotes longevity. Overall, UA-induced inter-organellar calcium signaling orchestrates the coordination between mitophagy, mitochondrial biogenesis and lysosomal activity, thereby contributing to increased healthspan and survival.

Our results suggest a model in which UA modulates inter-organellar communication through calcium dynamics to effectively coordinate mitophagy and mitochondrial biogenesis, thus maintaining a fully functional mitochondrial pool and extending lifespan (Figure 8). Specifically, UA triggers intracellular calcium elevation by promoting ER calcium release through ITR-1/ITPR/InsP3R and TMCO-1/F22B5.10/TMCO1 channels, assisted by EMC-3/EMC3, with subsequent mitochondrial calcium uptake via MCU-1/MCU. Disruption of calcium signaling, either through genetic ablation or chemical chelation, dramatically abolishes UA-induced mitophagy, impairing the beneficial effects of UA in muscle function and lifespan extension. Recent studies in *D. melanogaster* and MEFs have demonstrated that ITPR/InsP3R protein levels can be indirectly regulated by the PINK1/Parkin pathway, which in turn influences ER calcium release [43]. Notably, PINK-1/PINK1 is required for the lifespan-extending effects of UA in *C. elegans* [19]. Furthermore, the PINK1-Parkin pathway is involved in the regulation of mitochondrial calcium influx through VDACs and the MCU. Specifically, Parkin ubiquitinates MICU1 (mitochondrial calcium uptake protein 1), a positive regulator of MCU, leading to its degradation via the ubiquitin-proteasome system [99]. Interestingly, a very recently identified mitophagy inducer, kaempferol, has been shown to increase the rate of mitochondrial calcium uptake via the direct modulation of MCU-1/MCU, further highlighting the pivotal role of calcium homeostasis in mitophagy regulation. However whether kaempferol induces mitophagy and affects inter-organellar crosstalk similar to UA needs to be examined [100, 101]. This calcium flux facilitates DRP-1-mediated mitochondrial fission, priming dysfunctional mitochondria for autophagic degradation. Emerging evidence suggests that PINK1 phosphorylates DNM1L/DRP1 to regulate mitophagy and mitochondrial dynamics, however we did not detect any changes in DRP-1/DNM1L/DRP1 phosphorylation status in response to UA [66, 102]. These results further strengthen our findings and highlight the intricate interplay between calcium dynamics and mitophagy pathways in response to UA supplementation, ultimately contributing to its geroprotective effects.

Our multi-omics analysis further reveals that UA reprograms transcriptional and proteomic networks associated with calcium homeostasis, mitochondrial function and inter-organellar communication in nematodes and mammalian cells. UA altered the expression of genes and proteins involved in ER, mitochondrial, and lysosomal activity, indicating its broad influence on proteostasis, organelle integrity, and cellular stress responses. Importantly, both transcriptomic and proteomic data highlighted cytosolic calcium elevation as a central mechanism by which UA promotes mitophagy and maintains metabolic homeostasis, thus delaying age-related phenotypes. These adaptations reinforce the concept that UA induces global proteostatic remodeling, reestablishing functional organelle crosstalk to maintain energy production and proteostasis during ageing.

In line with these findings, previous studies have shown that UA reduces mitochondrial membrane potential, a mechanism proposed to stabilize PINK1 on the outer mitochondrial membrane and activate mitophagy [19]. Consistently, we observed that UA diminishes mitochondrial membrane potential in young *C. elegans*. However, during ageing, a period typically marked by a progressive decline in mitochondrial function, UA supplementation preserves mitochondrial membrane potential. This dual effect suggests that UA exerts a context-dependent regulation of mitochondrial function. Initially it induces a mild, transient depolarization to trigger mitophagy, followed by long-term stabilization of mitochondrial membrane potential to support mitochondrial bioenergetics. Such a biphasic mode of action may reflect UA’s ability to fine-tune mitochondrial quality control, balancing the clearance of damaged organelles with the preservation of healthy mitochondria via enhanced mitochondrial biogenesis, likely mediated by SKN-1/NFE2L2/Nrf2 activity. These findings support the notion that UA serves as a hormetic agent, eliciting adaptive stress responses that promote cellular resilience and organismal physiology over time.

Mechanistically, our findings reveal that UA-induced intracellular calcium elevation activates SKN-1/NFE2L2/Nrf2. This is aligned with a recent study in rodents demonstrating that UA and its structural analogs activate NFE2L2/Nrf2 to initiate an anti-inflammatory response in a colitis model [22]. NFE2L2/Nrf2 is well appreciated as a key regulator of mitochondrial biogenesis, with multiple studies highlighting its critical role in the maintenance of mitochondrial homeostasis [3, 67, 70–72]. The crosstalk between mitophagy and mitochondrial biogenesis is crucial for the maintenance of mitochondrial quality, particularly during ageing, where it ensures that cells not only degrade dysfunctional mitochondria, but also preserve a healthy mitochondrial pool to meet energy demands [67, 68]. In *C. elegans*, SKN-1/NFE2L2/Nrf2 is known to regulate both mitophagy and mitochondrial biogenesis [3]. Additionally, genetic studies in *C. elegans* suggested that UNC-43/CAMK2D directly targets DRP-1/DNM1L/DRP1 altering its phosphorylation status to modulate mitochondrial morphology in mechanosensory neurons during ageing [65]. Our findings demonstrate a nuanced role for UNC-43/CAMK2D and DRP-1/DNM1L/DRP1 in regulating mitochondrial quality control in response to UA. Under basal conditions, UNC-43 appears to limit DRP-1-mediated mitochondrial fission and mitophagy potentially through phosphorylation-dependent inhibition. However, in the context of UA treatment, UNC-43 primarily promotes SKN-1 activation and mitochondrial biogenesis rather than influencing DRP-1 localization or activity directly. This indicates distinct, yet interconnected roles for UNC-43/CAMK2D and DRP-1/DNM1L/DRP1, orchestrating a finely tuned response to maintain mitochondrial integrity and organismal health during ageing.

Importantly, the mechanisms uncovered here are evolutionarily conserved in mammalian systems. In human non-tumorigenic cells, UA enhances mitophagy, improves mitochondrial respiration, restores lysosomal function, and suppresses stress-induced premature senescence, all in a calcium-dependent manner. Notably, we found that UA upregulates cathepsins and enhances lysosomal function, consistent with recent findings that UA restores lysosomal activity in AD models [24]. Age-dependent lysosomal impairment is a common feature among eukaryotes [103, 104]. Congruently, we found that UA supplementation improves lysosomal activity in nematodes. In support, recent evidence suggested that nuanced role for UA nuanced role for influences p62-mediated lysophagy, highlighting its broader impact on the functionality of subcellular organelles as well as on inter-organellar wired communications to promote geroprotection [105]. By boosting mitochondrial-lysosomal signaling, UA ensures efficient mitochondrial turnover and maintains metabolic flexibility, further supporting the idea that UA can serve as a geroprotective agent capable of sustaining cellular health and resilience.

Our findings expand the role of mitophagy beyond the quality control of individual organelles to a broader concept that underscores mitophagy as a regulator of organellar connectivity and rejuvenation. Through calcium-mediated organelle rewiring, UA ensures that critical intracellular communication pathways are preserved, enabling cells to adapt to stress, maintain energy balance, and delay the onset of senescence. These results highlight an emerging paradigm, where restoring inter-organellar communication is as crucial as removing dysfunctional components to maintain organismal physiology. Given its conserved and multi-faceted actions, UA represents a promising therapeutic candidate for promoting healthy ageing and combating age-associated diseases characterized by impaired mitochondrial and lysosomal function. Future studies should explore how modulating calcium signaling and organelle contacts could synergize with mitophagy enhancers to optimize mitochondrial network remodeling and cellular rejuvenation.

Together, these findings suggest that by restoring inter-organellar communication networks and synchronizing mitochondrial degradation and renewal, cellular energy metabolism and stress resilience can be preserved during ageing. Our study highlights the therapeutic potential of targeting calcium signaling and mitophagy to rejuvenate organelle function and extend healthspan. These insights pave the way for new interventions aimed at combating age-associated diseases and promoting healthy ageing.

## Materials and methods

### *C. elegans* strains and maintenance

All *C. elegans* strains were maintained on NGM plates (3 g:L sodium chloride [NaCl; Sigma-Aldrich, 1.06404], 2.5 g:L peptone [AppliChem, A2206], 0.2 g:L streptomycin [AppliChem, A1852], 17 g:L Agar [AppliChem, A0949], 1 mM calcium chloride dihydrate [CaCl_2_; Sigma-Aldrich, 1.02382], 1 mM magnesium sulfate heptahydrate [MgSO_4_; Sigma-Aldrich, 1.05886], 25 mM potassium phosphate solution composed of 0.698 M potassium phosphate monobasic [KH_2_PO_4_; AppliChem 141,509] and 0.3014 M potassium phosphate dibasic [K_2_HPO_4_; AppliChem 141,512], pH 6.8, 5 mg:L cholesterol [AppliChem, A0807] and 5 mg:L nystatin [AppliChem, A3811]) seeded with *Escherichia coli* OP50 at 20°C using standard procedures [106]. The following strains were used in this study: N2: wild-type Bristol isolate, CZ19982: *mcu-1(ju1154)IV*, JT73: *itr-1(sa73)IV*, CU6372: *drp-1(tm1108)IV*, *unc-43(tm1605)IV*, CL2166: *dvIs19*[(pAF15)p_*gst-4*_GFP::NLS]III, CF1553: *muIs84*[(pAD76) p_*sod-3*_GFP + *rol-6(su1006)*], MAH240: *sqIs17*[p_*hlh-30*_HLH-30::GFP + *rol-6(su1006)*]; mitophagy in body wall muscles and neurons was monitored by using the strains IR1631: *unc-119(ed3)*; *Is001*[p_*myo-3*_TOMM-20::Rosella; *unc-119(+)*] and IR1864: N2; *Ex001*[p_*unc-119*_TOMM-20::Rosella; *rol-6(su1006)*], respectively; IR1155: N2; *Ex*[p_*let-858*_GCaMP2.0; *rol-6(su1006)*], HBR4: *unc-119(ed3)*; *goesIs3*[p_*myo-3*_GCaMP3.35; *unc-119(+)*], AQ3236: *ljSi2*[p_*mec-7*_GCaMP6.0 m::SL2::TagRFP; *unc-119(+)*]II and ATU2301: *goeIs3*[p_*myo-3*_SL1::GCamP3.35::SL2; *unc-119(+)*]; *aceIs1*[p_*myo-3*_mitoLAR-GECO; p_*myo-2*_RFP] were utilized to monitor cytosolic and mitochondrial calcium levels; muscle quality was evaluated in RW1596: *stEx30*[p_*myo-3*_GFP::MYO-3; *rol-6(su1006)*]; mitochondrial content in different tissues was monitored in SJZ216: *foxSi44*[p_*rgef-1*_TOMM-20::mKate2::HA::*tbb-2* 3’ UTR]I, SJZ47: *foxSi16*[p_*myo-3*_TOMM-20::mKate2::HA::*tbb-2* 3‘ UTR]I, SJZ328: *foxSi75*[p_*eft-3*_TOMM-20::mKate2::HA::*tbb-2* 3’ UTR]I and SJZ204: *foxSi37*[p_*ges-1*_TOMM-20::mKate2::HA::*tbb-2* 3’ UTR]I; mitochondrial network quality was assessed using SJ4103: *zcIs14*[p_*myo-3*_mtGFP]; the morphology of mitochondria, ER and lysosomes in the intestine was assessed using SJZ204: f*oxSi37*[p_*ges-1*_TOMM-20::mKate2::HA]I, VK2674: *vkEx2674*[p_*nhx-2*_CemOrange2::PISY-1; p_*myo-2*_GFP] and VK2688: *vkEx2688*[p_*nhx-2*_CemOrange2::CUP-5; p_*myo-2*_GFP], respectively; DRP-1 was monitored using EU2917: *drp-1*(*or1941*[DRP-1::GFP])IV and its association with mitochondria using KPA444: *drp-1*(*or1941*[DRP-1::GFP])IV; *foxSi16*[p_*myo-3*_TOMM-20::mKate2::HA]I, that was generated by crossing EU2917 with SJZ47. MAMs were studied in KPA427: *vkEx2674*[p_*nhx-2*_CemOrange2::PISY-1; p_*myo-2*_GFP]; zcIs17[p_*ges-1*_mtGFP] that was generated by crossing VK2674: *vkEx2674*[p_*nhx-2*_CemOrange2::PISY-1; p_*myo-2*_GFP] with SJ4143: zcIs17[p_*ges-1*_mtGFP). Additional strains generated for the purposes of this study were: *mcu-1(ju1154)IV; drp-1(tm1108)IV*, *drp-1(tm1108)IV; Ex001*[p_*unc-119*_TOMM-20::Rosella; *rol-6(su1006)*], *unc-43(tm1605)IV*; *Ex001*[p_*unc-119*_TOMM-20::Rosella; *rol-6(su1006)*] and *unc-43(tm1605)IV*; *dvIs19*[(pAF15)p_*gst-4*_GFP::NLS]III. Primers used for genotyping are listed in Table S1. For RNAi experiments, synchronous animal populations of each strain were generated either by bleaching or by egg laying on freshly made RNAi plates, seeded with HT115(DE3) bacteria, which had already been transformed with either the empty RNAi vector pL4440 or the indicated RNAi construct, supplemented with 2 mM isopropyl β-d-1 thiogalactopyranoside/IPTG (PanReacAppliChem, A1008). Constructs for *itr-1*, *pink-1, pdr-1, dct-1, drp-1*, *skn-1* and *unc-43* silencing were generated in previous studies [3,30]. Clones for *emc-3* and *tmco-1* were resourced from the Ahringer library [107]. The RNAi construct for *mcu-1* was a gift by Prof. Tavernarakis. Chemical compounds were added directly to NGM plates at the following final concentrations: Urolithin A (UA; Santa Cruz Biotechnology, sc -475,514): 50 μΜ, ethylene glycol-bis(2-aminoethylether)-N,N,N’,N’-tetraacetic acid (EGTA; Sigma-Aldrich, E4378): 10 mM, nicotinamide mononucleotide (NMN; gift from Dr. David A Sinclair, Department of Genetics, Harvard Medical School, Boston, USA): 5 mM, paraquat (PQ; methyl viologen dichloride hydrate; Sigma-Aldrich 856,177): 10 mM, N-acetylcysteine (NAC; Sigma-Aldrich, 1.12422): 10 mM, tetramethylrhodamine ethyl ester perchlorate (TMRE; Sigma-Aldrich 87,917): 0.15 μΜ, LysoSensor Green DND-189 (Invitrogen, L7535): 10 μΜ.

### *C. elegans* microscopy and image analysis

Images of GCaMP3.35/mitochondrial LAR-GECO, GFP-fluorescent mitochondria or DRP-1::GFP/mKate2-tagged mitochondria in muscles, and fluorescently tagged mitochondria, ER or lysosomes in the intestine were captured on a Zeiss LSM900 Confocal Microscope, using a 40x magnification. All other images of *C. elegans* strains were acquired using the EVOS M7000 Imaging System (Invitrogen by Thermo Fisher Scientific). Worms were immobilized in drops of 40 mM tetramisole hydrochloride (Sigma-Aldrich, T1512). Images of the same experiment were acquired under the same conditions. Quantification of images was performed with Fiji (ImageJ) software and statistical analysis was performed using GraphPad Prism 9.0.0 (GraphPad Software, San Diego, California USA, www.graphpad.com). The structural characteristics of intestinal organelles were determined in the most distal cells of the tissue. The quality of mitochondrial network in muscles was assessed based on the shape and connectivity of organelles and was scored as either tubular, fragmented or mediocre. To quantify the area of MAMs, double fluorescent images were captured at the most distal part of the intestine in KPA427 animals, and each channel was used to generate a binary mask. Noise was removed from both masks by applying a median filter (0.5 radius) and the one corresponding to mitochondria was used to create outlines that represent mitochondrial surface. A third mask that corresponds to MAMs was generated by isolating common pixels between the ER and the mitochondrial surface masks, using the “AND” function of the image calculator. The area of MAMs was expressed as the % of total mitochondrial surface. Two areas of 10.45x10.45 μm were analyzed for each nematode and their average was plotted. For DRP-1::GFP, analysis was performed in individual muscle cells. Double fluorescent images of KPA444 animals were acquired and the mKate2 channel was used to identify the area that corresponds to mitochondria and the limits of each muscle cell. The number of DRP-1::GFP particles was determined using the Find Maxima function of Fiji (prominence of 10). Images of p_*gst-4*_GFP, p_*sod-3*_GFP, p_*myo-3*_GCaMP3.35, p_*myo-3*_TOMM-20::mKate2, p_*eft-3*_TOMM-20::mKate2, p_*ges-1*_TOMM-20::mKate2 animals and worms treated with LysoSensor green or TMRE were captured under a 4x magnification and total pixel intensity was measured in whole bodies. LysoSensor Green was diluted in drops of M9 buffer and worms were vitally stained for 1 hour, followed by 1 hour recovery in NGM plates. The lysosomal activity was calculated by measuring the total pixel intensity of entire worms, with a higher GFP signal indicating increased activity. p_*rgef-1*_TOMM-20::mKate2 images were captured with a 10x objective lens and pixel intensity was measured only in the head area. To monitor muscle and neuronal mitophagy, images were acquired on both red and green channels at the head region of p_*myo-3*_TOMM-20::Rosella and p_*unc-119*_TOMM-20::Rosella expressing animals, respectively, using a 10x magnification. Analysis was performed as previously described [37]. Mitophagic flux was quantified in *C. elegans* expressing the TOMM-20::Rosella reporter by analyzing the ratio of red-only to total mitochondrial particles using Fiji software. Images from both green (GFP) and red (DsRed) fluorescence channels were split, and regions of interest (ROIs), such as the head region, were selected and added to the ROI manager. Background subtraction (50%) was applied separately to both channels. A consistent threshold was applied to each channel to isolate mitochondrial particles. Particle analysis was then performed for each channel. To assess colocalization, the Image Calculator tool in Fiji was used to compute the overlap between red and green signals (AND operation). The resulting image showed only colocalized (GFP^+^ DsRed^+^) mitochondria. Mitophagic flux was calculated as the ratio of red-only puncta (non-colocalized red particles) to total red particles, representing the fraction of mitochondria undergoing mitophagy (i.e., mitochondria delivered to the lysosome where GFP is quenched but DsRed persists). Images of p_*hlh-30*_HLH-30::GFP animals head region were acquired using a 10x objective lens and the degree of nuclear localization was judged based on the brightness of the nuclei. Images of p_*myo-3*_GFP::MYO-3 animals were captured using a 20x magnification and the quality of body wall muscles was scored as good, mediocre or severe based on the connectivity, breaks and compactness of the myofilaments. Images of p_*let-858*_GCaMP2.0 were captured using 20x magnification under a green channel. Touch receptor neurons of p_*mec-7*_::GCaMP6.0 m::SL2::TagRFP animals were acquired on both a green and a red channel under a 40x objective lens. The ratio of GFP:RFP was calculated to reduce movement artifacts [108].

### Thrashing assays

For thrashing assays, 1-day, and 8-day adult nematodes were placed in a 10 μl drop of M9 buffer (22 mM potassium di-hydrogen phosphate [KH_2_PO_4_; AppliChem 141,509], 42.3 mM sodium phosphate dibasic [Na_2_HPO_4_; Sigma-Aldrich, 1.06586], pH 7, 86 mM sodium chloride [NaCl; Sigma-Aldrich, 1.06404] and 1 mM magnesium sulfate heptahydrate [MgSO_4_;Sigma-Aldrich, 1.05886]) on a microscope slide and observed under the stereoscope at room temperature (21–23°C). The number of thrashes/body bends for each individual nematode was counted every time the nematode moved in the opposite direction of the previous body bend. The thrashes/body bends were counted for 20 s. For each condition, 20 individual animals were examined, and each one was assessed at three independent times.

### Lifespan assays

All lifespan assays were performed at 20°C. Semi-synchronous animal populations were generated by egg laying and progeny were allowed to grow under appropriate, defined conditions. 20–25 larval stage 4 (L4) animals were reared per NGM plate, for a total of 100–150 individuals per experiment. For RNAi experiments, L4 worms were placed on plates seeded with HT115(DE3) *E. coli* bacteria, transformed with either the pL4440 plasmid vector (Fire Lab *C. elegans* Vector Kit) or the specific RNAi plasmid construct, and supplemented with 2 mM isopropyl β-d-1 thiogalactopyranoside (IPTG). Animals were transferred to fresh plates every 2–4 days thereafter and examined daily for touch-provoked movement and pharyngeal pumping, until death. Worms that died owing to internally hatched eggs, an extruded gonad or desiccation due to crawling on the edge of the plates were censored and incorporated as such into the data set. Survival curves were created using the product limit method of Kaplan and Meier. The log rank (Mantel – Cox) test was used to evaluate differences between survivals and determine *p* values. Statistical analysis was performed using GraphPad Prism 9.0.0 (GraphPad Software, San Diego, California USA, www.graphpad.com). Detailed data from lifespan assays are presented in Table S2 and S3.

### Cell lines and cell culture conditions

Human foreskin fibroblasts (BJ; ATCC, CRL-2522) and human umbilical vein endothelial cells (HUVECs; ATCC, CRL-1730) were obtained from the American Tissue Culture Collection. BJ were cultured in Dulbecco’s Modified Eagle’s Medium (DMEM; Thermo Fisher Scientific 11,965,092) supplemented with 10% (v:v) fetal bovine serum (FBS; Sigma-Aldrich, F7524) and 1% (v:v) non-essential amino acids and HUVECs were maintained in Medium 199 (M199; Invitrogen 41,150,020) supplemented with 20% (v:v) FBS and 1% large vessel endothelial supplement (LVES; Gibco Life Technologies, A1460801). Mouse embryonic fibroblasts (MEFs; gift by National Institutes of Health, USA) were cultured in Dulbecco’s Modified Eagle Medium (Thermo Fisher Scientific 31,966,021) supplemented with 10% FBS and 1% penicillin-streptomycin (Thermo Fisher Scientific 15,140,122), in a humidified 37°C incubator with 5% CO_2_. All cells were maintained in conditions of 5% CO_2_, 95% humidity and 37°C. In all experimental procedures applied, cells were subcultured by using a trypsin-EDTA solution (Thermo Fisher Scientific, R001100). HMC3 cells (ATCC, CRL-3304) were cultured in Minimum Essential Medium Eagle (Merck, M2279) containing 10% FBS and 1% GlutaMAX (Thermo Fisher Scientific 35,050,061) and treated 1 week with or without UA. Then cells were reseeded on 96-well plates for reading fluorescence on a Flexstation 3 plate reader (Molecular Devices) using filter excitation 490-nm and Emission 515-nm. The Calbryte^TM^ 520 (Nordic Biosite, ABD-20651) was prepared and used following the instruction manual. In short, cells were washed in HBSS (VWR, HYCLSH30268.01), then 10 nM Calbryte^TM^ 520 in HBSS was incubated with wells with or without 10 μM UA and or 1 mM EGTA for 45 min at room temperature. Buffer was exchanged to HBSS again before plates were read.

### Cell viability assay

Cells were plated in flat-bottomed 96-well microplates and the next day they were incubated with different concentrations of UA for 24 h. Afterwards, the medium was replaced by 3-(4,5-dimethylthiazol-2-yl)-2,5-diphenyltetrazolium bromide (MTT; Sigma-Aldrich 475,989) dissolved at a final concentration of 1 mg:mL in serum-free, phenol red-free medium. The formed formazan crystals were then dissolved by isopropanol and the absorbance of the solution was measured at 570-nm wavelength. Survival of control cells was arbitrarily set to 100%.

### Measurement of cathepsins enzymatic activities

For measuring cathepsins activity cells were lysed in an extraction buffer (1 mM dithiothreitol and Tris 50 mM, pH 4.0) and the lysates were cleared at 17,000 g for 20 min at 4°C. Following protein content measurement with Bradford assay, 5 μg of protein were incubated in the reaction buffer (50 mM sodium acetate, 8 mM cysteine-hydrochloride [Sigma-Aldrich, C1276], 1 mM EDTA, pH 4.0) containing the fluorogenic substrate Z-RR-AMC (Enzo Life Sciences, Inc., BML-P137) for 30 min at 37°C. Fluorescence was measured in a Spark multimode microplate reader (Tecan Trading AG, Männedorf, Switzerland) at excitation and emission wavelengths of 350- and 440-nm, respectively.

### Measurement of reactive oxygen species (ROS)

For the assessment of ROS production, cells were incubated with 10 μM CM-H_2_DCFDA dye (Thermo Fisher Scientific, C6827) in PBS (Thermo Fisher Scientific 10,010,023) for 30 min at 37°C in the dark. Following dye removal, cells were incubated for 10 min with PBS and then lysed in Nonidet *p*-40 lysis buffer (1% Nonidet *p*-40 [NP-40; Thermo Fisher Scientific 85,124], 150 mM NaCl, and 50 mM Tris, pH 8.0). The cells were cleared by centrifugation at 19,000 g for 10 min at 4°C. The supernatant was diluted 1:4 (v:v) in ddH2O, and fluorescent dichlorodihydrofluorescein was measured in a Spark multimode microplate reader (Tecan Trading AG, Männedorf, Switzerland) at excitation, 490-nm, and emission, 520-nm.

### Intracellular calcium measurement

For the calcium detection, cells were incubated with 10 μM Fluo-4 AM dye (Invitrogen, Thermo-Fisher, F14201) in PBS for 30 min at 37°C in the dark. Following dye removal, cells were incubated for 10 min with PBS and then, either were stained with DAPI and viewed at the NIKON C1 CLSM or the produced fluorescence was measured using the Infinite 200 Tecan microtiter-plate photometer (Tecan Trading AG, Switzerland) at excitation and emission wavelengths of 485- and 535-nm, respectively.

### Preparation of cell extracts and immunoblotting analysis

For immunoblotting studies cells were lysed on ice in NP-40 lysis buffer (150 mM NaCl, 1% NP-40, 50 mM Tris, pH 8.0) containing protease (Sigma-Aldrich, P8340) and phosphatase (Sigma-Aldrich, P2850) inhibitors and lysates were immediately cleared with centrifugation for 15 min at 17,000 g (4 °C). The supernatant was collected and following the determination of protein concentration by Bradford (Bio-Rad Laboratories, Inc., 5000006), samples were fractionated by SDS-PAGE and analyzed by immunoblotting. Primary and horseradish peroxidase-conjugated secondary antibodies were applied for 1 h at room temperature and immunoblots were developed using an enhanced chemiluminescence reagent kit (Clarity^TM^ Western ECL Substrate; Bio-Rad Laboratories, Inc., 1705061). Immunoblots quantitation was performed by scanning densitometry and ImageJ software. Primary antibody against LC3B (2775) was purchased from Cell Signaling Technology, Inc. Primary antibody against GAPDH (sc‐25778), as well as horseradish peroxidase‐conjugated secondary antibodies (sc-2357), were purchased from Santa Cruz Biotechnology.

### Mitophagy assessment in cell cultures

For the detection of mitophagy, Mitophagy Detection Kit (Dojindo Molecular Technologies, MD01) was used. Cells were treated according to the manufacturer’s recommendations. In brief, cells were washed twice with serum-free DMEM (Thermo Fisher Scientific 21,063,029) and afterwards incubated at 37°C for 30 min with 100 nmol:L Mitophagy Dye diluted in serum-free DMEM. After this incubation cells were again washed twice with serum-free DMEM followed by the addition of complete DMEM. Samples were viewed at a NIKON C1 Confocal Laser Scanning Microscope (CLSM).

### Seahorse cellular stress assays

To evaluate the changes in mitochondrial function, Seahorse XFp Cell MitoStress Test (Agilent Technologies 103,015–100) was performed according to the manufacturer’s protocol. In brief, on the day of the assay, the cell culture growth medium was replaced with low-buffered pH 7.4 DMEM supplemented with glutamine (2 mM; Thermo Fisher Scientific 25,030,081), glucose (10 mM; Thermo Fisher Scientific, A2494001) and sodium pyruvate (1 mM; Thermo Fisher Scientific 11,360,070). The cell culture microplate was incubated in a non-CO_2_ incubator at 37°C for 1 h before the assay. The seahorse compounds were prepared in assay media and injected into the injection ports.

### Induction of premature senescence by H_2_O_2_ and SA-GLB1/β-gal staining

For the induction of premature senescence, proliferating cells were exposed to the sub-cytotoxic concentration of 300 μM H_2_O_2_ (three exposures of 48 h each), as previously described [109]. Senescent cells were stained with GLB1/β-galactosidase staining as described previously [110]. Briefly, cells were washed with PBS and fixed in 2% formaldehyde in PBS for 5 min. Following fixation cells were washed with PBS and were then stained (in the absence of CO_2_) for 12–16 h at 37°C in staining solution (150 mM NaCl, 2 mM MgCl_2_, 5 mM K_3_Fe(CN)_6_ [Sigma-Aldrich 244,023], 5 mM K_4_[Fe(CN)_6_]·3 H_2_O [Sigma-Aldrich, P3289], 40 mM citric acid/sodium phosphate, pH 6.0 containing 1 mg/ml 5-bromo-4-chloro-3-indolyl-β-d-galactoside [Thermo Fisher Scientific, B1690]). Cells were viewed under phase contrast optics in a TS-100F NIKON inverted microscope and at least 10 optical fields were used to score positively stained cells.

### RNA isolation, RT-qPCR and RNAseq

*C. elegans* total RNA was prepared from frozen worm pellets of OP50-fed N2 animals treated with 50 μM UA, using Nucleozol (Macherey-Nagel 740,404). Four separate populations were allowed to grow in the presence of UA and 100–150 animals were harvested at day 1 of adulthood and treated independently. Additional populations propagated in parallel under the same conditions without UA were included in each sample group as the control. The quality and quantity of RNA samples were determined using BioTek Cytation 5 reader (Agilent). Reverse transcription was carried out with iScript RT cDNA Synthesis KIT (Bio-Rad) and quantitative PCR was performed using KAPA SYBR FAST Universal Kit (Roche, KK4602) in the QuantStudio 5 Real-Time PCR system (Applied Biosystems). Relative amounts of mRNA were determined using the comparative Ct method for quantification and each sample was independently normalized to its endogenous reference gene (*ama-1*). Gene expression data are presented as the mean fold change ± SEM of all biological replicates relative to the indicated control. Statistical analysis was performed as indicated on each occasion. Primers used for qRT-PCR are listed in Table S1. For RNAseq studies, libraries were prepared using the DNBSEQ Eukaryotic Stranded Transcriptome library – Stranded mRNA Library (performed by BGI Genomics’ RNA sequencing service; www.bgi.com). Library preparation started with mRNA enrichment and purification (using oligo dT beads to enrich mRNA with poly A tail). The RNA was fragmented, and first-strand cDNA was generated using random N6-primed reverse transcription, followed by a second-strand cDNA synthesis with dUTP instead of dTTP. The synthesized cDNA underwent end-repair followed by 3’ adenylation. Adaptors were ligated to the ends of these 3’ adenylated cDNA fragments. Prior to PCR amplification, the dUTP-marked strand was selectively degraded by UNG/UDG (uracil DNA glycosylase). The remaining strand was amplified to generate a cDNA library suitable for sequencing. Multiple rounds of PCR amplification were performed to enrich the purified cDNA template using PCR primer. The PCR product was denatured by heat and the single-stranded DNA was cyclized by splint oligo and DNA ligase. The DNA nanoball was synthesized and sequenced on DNBSEQ (DNBSEQ Technology) platform (BGI Genomics). Total RNA from cells was extracted using RNAiso plus (Takara Bio Inc., 9108) and quantified with a BioSpec-nano spectrophotometer (Shi-madzu Inc., Kyoto, Japan). Subsequently, 1 μg RNA was converted to cDNA using the FastGene Scriptase II cDNA Kit (NIPPON Genetics, LS63). To carry out Quantitative Real-Time PCR analyses, the 5x HOT FIREPol® EvaGreen® qPCR Supermix (Solis BioDyne, 08–36) and PikoRe-alTM Real-Time PCR System (Thermo Fisher Scientific, Waltham, MA, USA) were used. Primers were designed using the primer-BLAST tool (http://www.ncbi.nlm.nih.gov/tools/primer-blast/).

### RNAseq analysis

RNAseq reads were subjected to quality control (QC) to determine whether the sequencing data is suitable for subsequent analysis. Filtered clean reads were aligned to Genome assembly WBcel235 (GCF_000002985.6_WBcel235) using HISAT. FeatureCounts v.1.6.2 was used to generate a matrix of mapped fragments per RefSeq annotated gene. Analysis for differential expressed genes (DEGs) between the UA and control samples was performed using the R package DESeq2 v.1.16.1 with a threshold of *p* value < 0.05 and |FC| > 1.2. PCA (principal components analysis) was completed by R package factoextra v1.0.7. Volcano plots and dot plots were achieved with the R package ggplot2 v.3.5.1. Heatmap of DEG expression was visualized using the R package pheatmap v1.0.12. Gene ontology (GO) enrichment analysis was determined by Gene Ontology: http://geneontology.org.

### Mitochondrial isolation and immunoblotting in *C. elegans*

Mitochondrial and cytosolic fractions were isolated as previously described [73], with minor modifications. Briefly, approximately 600 *C. elegans* were collected in M9 buffer and homogenized with an equal volume of ice-cold isolation buffer (0.32 M sucrose [Apollo Scientific, BIS3391], 10 mM EDTA, 10 mM Tris-HCl, pH 7.3) supplemented with 2% (w:v) BSA (Sigma-Aldrich, A9418). The homogenate was passed through a 10-μm filter and centrifuged at 2,200 *g* for 10 min at 4°C. The resulting mitochondrial pellet was washed with BSA-free isolation buffer and resuspended in 40 μL of 1× SDS-PAGE sample buffer. The supernatant (cytosolic fraction) was retained and mixed with 5× SDS-PAGE sample buffer.

Both fractions were boiled and analyzed by SDS-PAGE followed by immunoblotting, as previously described [73]. Primary antibodies used were: anti-GFP (Santa Cruz Biotechnology, sc-9996), anti-ACT-2 (Cell Signaling Technology, 4967), and anti-HSP-60 (Developmental Studies Hybridoma Bank, AB_10572252). HRP-conjugated secondary antibodies were incubated for 1 h at room temperature. Immunoreactive signals were detected using an enhanced chemiluminescence (ECL) kit (GE Healthcare Amersham, RPN2108). Band intensities were quantified using Fiji (ImageJ) software.

### LC-MS/MS analysis

For total proteomic analysis *C. elegans* samples (pool 500 worms) were lysed in a lysis buffer consisting of 4% SDS, 0.1 M DTT, 0.1 M Tris, pH 7.4. The samples were subjected to heating for 3 min at 99°C followed by a centrifugation step for 15 min at 17,000 g.

For DRP-1 immunoprecipitation analysis, four biological replicates and three to four technical replicates per genotype were utilized. Briefly, *C. elegans* expressing DRP-1::GFP, with or without UA treatment, were harvested and homogenized in a lysis buffer composed of 50 mM Tris-HCl, pH 7.4, 150 mM NaCl, 1 mM EDTA, and 1% Triton X-100 (Sigma-Aldrich, T8787), supplemented with protease and phosphatase inhibitors; to eliminate nonspecific protein interactions, worms expressing GFP alone were used as controls. Following centrifugation, the resulting supernatants were incubated overnight at 4°C with anti-GFP-conjugated magnetic beads (Cell Signaling Technology, 67090S) under continuous agitation. After incubation, the beads were subjected to five sequential washes. Protein elution was performed under acidic conditions using 0.1 M glycine-HCl (pH 3.5).

Samples were processed according to the Sp3 protocol [104] including an alkylation step in 200 mM iodoacetamide (Thermo Fisher Scientific, AC122270050). 20 μg of beads (1:1 mixture of hydrophilic and hydrophobic SeraMag carboxylate-modified beads; GE Healthcare Greece 45,152,105,050,250 and 65,152,105,050,250) were added to each sample in 50% ethanol. Protein clean-up was performed on a magnetic rack. The beads were washed twice with 80% ethanol and once with 100% acetonitrile (Fisher Chemical, A955–500). The captured-on beads proteins were digested overnight at 37°C under vigorous shaking (1200 rpm, Eppendorf Thermomixer) with 1 μg Trypsin/LysC (MS grade; Promega, V5071) prepared in 25 mM ammonium bicarbonate, pH 7.8. Next day, the supernatants were collected, and the peptides were purified using a modified Sp3 clean up protocol and finally solubilized in the mobile phase A (0.1% formic acid in water), sonicated and the peptide concentration was determined through absorbance at 280 nm measurement using a nanodrop instrument.

Samples were run on a liquid chromatography tandem mass spectrometry (LC-MS/MS) setup consisting of a Dionex Ultimate 3000RSLC online with a Thermo Q Exactive HF-X Orbitrap mass spectrometer. Peptidic samples were directly injected and separated on an 25-cm-long analytical C18 column (PepSep, 1.9 μm^3^ beads, 75 µm ID; Bruker Daltonics 1,893,477) using a 90 min-long run, starting with a gradient of 7% Buffer B (0.1% formic acid in 80% acetonitrile) to 28% Buffer B for 63 min and followed by an increase to 36% in 7 min and a second increase to 95% in 0.5 min and then kept constant for equilibration at 7% Buffer B for 14.5 min. A full MS was acquired in profile mode using a Q Exactive HF-X Hybrid Quadrupole-Orbitrap mass spectrometer, operating in the scan range of 375–1400 m:z using 120 K resolving power with an AGC of 3x 10^6^ and max IT of 60 ms followed by data independent analysis using 8 Th windows (39 loop counts) with 15K resolving power with an AGC of 3x 10^5^ and max IT of 22 ms and a normalized collision energy (NCE) of 26. Each biological sample was analyzed in three technical replicas.

### Proteomics data analysis

Orbitrap raw data was analyzed in DIA-NN (Data-Independent Acquisition by Neural Networks) (Demichev) through searching against the reference proteomes of *C. elegans* (UP000001940_6239) and *E. coli* (UP000000625_83333), both retrieved from Uniprot database on December ‎20, ‎2023, in the library free mode of the software, allowing up to two tryptic missed cleavages. A spectral library was created from the DIA runs and used to re-analyze them. DIA-NN default settings have been used with oxidation of methionine residues and acetylation of the protein N-termini set as variable modifications and carbamidomethylation of cysteine residues as fixed modification. N-terminal methionine excision was also enabled. In case of the analysis of the enriched DRP-1 through its immunoprecipitation, the phosphorylation of serines/threonines and tyrosines were set as potential variable modification. The match between runs (MBR) feature was used for all analyses and the output (precursor) was filtered at 0.01 FDR and finally the protein inference was performed on the level of genes using only proteotypic peptides. The software directLFQ was applied for additional sample normalization and the calculation of protein intensities (Ammar). The generated results were processed statistically and visualized in the Perseus software 1.6.15.0 (Tyanova). Values were log_2_ transformed, a threshold of 70% of valid values in at least one group was applied and the missing values were replaced from normal distribution. For statistical analysis, Student’s t-test was performed, and permutation-based FDR was calculated. Analysis for DEPs was conducted in a total of 4603 proteins detected using Student’s t-test with a threshold of *p* value < 0.05 between the UA and control samples. PCA (principal components analysis) was completed by R package factoextra v1.0.7. Volcano plots and dot plots were achieved with the R package ggplot2 v.3.5.1. Gene ontology (GO) enrichment analysis was determined by Gene Ontology: http://geneontology.org.

### GSVA analysis

Gene set variation analysis (GSVA) was carried out to estimate sample-wise GO pathway activity variation using the GSVA R package version 1.50.

### Statistics and reproducibility

GraphPad Prism 9.0.0 (GraphPad Software, San Diego, California USA, www.graphpad.com) was used for statistical analyses. Statistical details of experiments can be found in the figures’ legends. The log-rank (Mantel – Cox) method was used to compare survival curves. Mean values were compared using unpaired t-tests. For all experiments, *p* values < 0.05 were considered significant. Data distribution was assumed to be normal, but this was not formally tested. No statistical method was used to predetermine the sample size, but our sample sizes are similar to those reported in previous publications using similar procedures [3,19,20,111,112]. The investigators were not blinded to allocation during the experiments and/or data collection and outcome assessment or analysis. All experiments with objective measurements (such as microscopy, thrashing assays and lifespan assays) were also performed blinded by other members of the laboratory.

### Data availability

The authors declare that all data supporting the findings of this study are available within the paper and its supplementary information files. The RNA sequencing data have been deposited to GEO repository with the dataset identifier GSE279559. The mass spectrometry proteomics data have been deposited to the ProteomeXchange Consortium via the PRIDE [113] partner repository with the dataset identifier PXD056571.

## Supplementary Material

Supplementary_Data_file_autophagy_R5_KP.docx

## Data Availability

All data supporting the findings of the current study are available within the paper and its Supplementary Material. The datasets generated, used and analyzed during this study and the respective materials are available from the corresponding authors upon request.
