# Supplementary material for: Urolithin Α modulates inter-organellar communication via calcium-dependent mitophagy to promote healthy ageing
Source: Autophagy. 2025 Sep 29;21(12):3097–122. doi: 10.1080/15548627.2025.2561073 (PMC12758194; doi:10.1080/15548627.2025.2561073)
Supplement: Supplementary_Data_file_autophagy_R5_KP.docx [file KAUP_A_2561073_SM8628.docx]

| **Table S1.** List of primers used in this study. | | |
| --- | --- | --- |
| **Oligo name** | **Sequence (5-‘3’)** | **Application** |
| *mcu-1(ju1154)_F* | ATGAGGAATGGCCGATGCTTG | Tracking of *ju1154* allele |
| *mcu-1(ju1154)_R* | GCTAACGGGAAGATGCTGGAATAG |  |
| *mcu-1_RT1* | CTTCCGTCAAGAAATGAGCCTTG | qRT-PCR |
| *mcu-1_RT2* | CGTCGAGTTGGCGGAGTTTATC |  |
| *unc-43(tm1605)_F* | AAAGAAGCTATCCGCTCGTGAC | Tracking of *tm1605* allele |
| *unc-43(tm1605) _R* | CCAAATTTTCTCATTCGCGC |  |
| *drp-1(tm1108)_F* | ATGGAACACTTCTCACAGATGATTGG | Tracking of *tm1108* allele |
| *drp-1(tm1108)_R* | TCCTGGTACCGGTTGTAGCTGC |  |
| *itr-1_RT1* | GATTCTTGAGCACACTTGGATTGG | qRT-PCR |
| *itr-1_RT2* | AGCAACTTTCAGCTTATTCATCAGATC |  |
| *sca-1_RT1* | CAAAGACGCCAATGAGGTGTG | qRT-PCR |
| *sca-1_RT2* | TCACTGCTTCTGTCTGATCTTCG |  |
| *ama-1_RT1* | GCTATGGTGCCGAGACAAC | qRT-PCR  (Reference gene) |
| *ama-1_RT2* | CCAGGAATGATAAGCGAGAAGAC |  |
| *emc-3 _RT1* | GGAGAATATAGCCGACGGACAATAC | qRT-PCR |
| *emc-3 _RT2* | CGACGACGATCATTGGGATCATAT |  |
| *tmco-1_RT1* | ACAAAAGACTGAAAGCTGATATGGAC | qRT-PCR |
| *tmco-1_RT2* | CAGTCATGTCTTCGCCAATCAAG |  |
| *BECN1_F* | AACCAGATGCGTTATGCCCA | qRT-PCR |
| *BECN1_R* | TCCATTCCACGGGAACACTG |  |
| *p62_F* | CATCGGAGGATCCGAGTGTG | qRT-PCR |
| *p62_R* | TTCTTTTCCCTCCGTGCTCC |  |
| *ATG5_F* | GGCCATCAATCGGAAACTCA | qRT-PCR |
| *ATG5_R* | CCTAGTGTGTGCAACTGTCCA |  |
| *NRF2_F* | CATCCAGTCAGAAACCAGTGG | qRT-PCR |
| *NRF2_R* | GCAGTCATCAAAGTACAAAGCAT |  |
| *NQO1_F* | AGCAGACGCCCGAATTCAAA | qRT-PCR |
| *NQO1_R* | AGAGGCTGCTTGGAGCAAAA |  |
| *TXNRD1_F* | TTGGAGTGCGCTGGATTTCT | qRT-PCR |
| *TXNRD1_R* | TTTGTTGGCCATGTCCTGGT |  |
| *p21_F* | GACCATGTGGACCTGTCACT | qRT-PCR |
| *p21_R* | CTTCCTGTGGGCGGATTAGG |  |
| *GAPDH_F* | CCACATCGCTCAGACACCAT | qRT-PCR |
| *GAPDH_R* | CCATGGGTGGAATCATATTGGAAC |  |

| **Table S2.** Lifespan analysis of UA-treated animals in OP50-seeded NGM plates. | | | | | | |
| --- | --- | --- | --- | --- | --- | --- |
| **#** | **Stain** | **Treatment** | **Median/Max** | **N^1^ (T/C)** | **p-value (vs Ctrl)** | **p-value (vs wt)** |
| 1 | N2 | - | 17/23 | 39/0 | 0.2597 | - |
|  |  | UA | 18/24 | 70/4 |  |  |
|  |  | EGTA | 15/19 | 65/0 | 0.7423 |  |
|  |  | UA + EGTA | 15/20 | 49/1 |  |  |
| 2 | N2 | - | 14/16 | 55/1 | <0.0001(****) | - |
|  |  | UA | 16/25 | 77/9 |  |  |
|  |  | EGTA | 13/19 | 89/18 | 0.3987 |  |
|  |  | UA + EGTA | 13/22 | 102/30 |  |  |
| 3^◊^ | N2 | - | 17/25 | 94/5 | 0.0078(**) | - |
|  |  | UA | 18/28 | 90/10 |  |  |
|  |  | EGTA | 17/24 | 95/3 | 0.9733 |  |
|  |  | UA + EGTA | 17/25 | 97/4 |  |  |
| 4 | N2 | - | 17/23 | 134/8 | - | - |
|  |  | UA | 19/24 | 90/7 | 0.0008(***) | - |
|  | *mcu-1*  *(ju1154)* | - | 15/20 | 134/4 | - | <0.0001(****) |
|  |  | UA | 14/20 | 148/18 | 0.3680 | <0.0001(****) |
|  | *itr-1*  *(sa73)* | - | 14/18 | 140/14 | - | <0.0001(****) |
|  |  | UA | 14/19 | 143/19 | 0.3480 | <0.0001(****) |
| 5^◊^ | N2 | - | 21/27 | 132/26 | - | - |
|  |  | UA | 24/29 | 86/2 | 0.0002 | - |
|  | *mcu-1*  *(ju1154)* | - | 18/24 | 127/6 | - | <0.0001(****) |
|  |  | UA | 18/25 | 120/3 | 0.6989 | <0.0001(****) |
|  | *itr-1*  *(sa73)* | - | 20/24 | 140/2 | - | <0.0001(****) |
|  |  | UA | 19/26 | 135/0 | 0.4834 | <0.0001(****) |
| All experiments were carried out at 20°C in OP50-seeded NGM plates supplemented with the indicated chemical (treatment).  *p<0.05, **p<0.01, ***p<0.001, ****p<0.0001 - Log-rank (Mantel-Cox) test  ^1^T = total/C = censored.  ^◊^marks data shown in figures. | | | | | | |

| **Table S3.** Lifespan analysis of UA-treated animals exposed to RNAi. | | | | | | | | |
| --- | --- | --- | --- | --- | --- | --- | --- | --- |
| **#** | **Stain** | **RNAi** | **Treatment** | **Median/Max** | **N^2^ (T/C)** | **vs Empty Vector** | **vs Control** | **vs N2** |
| 1^◊^ | N2 | Empty  Vector | Control | 18/26 | 138/18 | - | - | - |
|  |  |  | UA (50 μM) | 22/26 | 146/10 |  | <0.0001(****) |  |
|  |  | *itr-1*  *(RNAi)* | Control | 12/20 | 89/11 | <0.0001(****) | - |  |
|  |  |  | UA (50 μM) | 11/18 | 119/16 | <0.0001(****) | 0.0334(*) |  |
|  | *mcu-1*  *(ju1154)* | Empty  Vector | Control | 18/25 | 130/12 | - | - | 0.8858(ns) |
|  |  |  | UA (50 μM) | 21/27 | 141/3 |  | 0.0001(****) | 0.0113(*) |
|  |  | *itr-1*  *(RNAi)* | Control | 10/20 | 65/10 | <0.0001(****) | - | 0.0495(*) |
|  |  |  | UA (50 μM) | 10/16 | 84/13 | <0.0001(****) | 0.2915(ns) | 0.0032(**) |
|  | *drp-1* | Empty  Vector | Control | 18/26 | 133/17 | - | - | 0.8400(ns) |
|  |  |  | UA (50 μM) | 19/29 | 138/13 |  | 0.0354(*) | 0.0066(**) |
|  |  | *itr-1*  *(RNAi)* | Control | 14/24 | 90/8 | <0.0001(****) | - | 0.0048(**) |
|  |  |  | UA (50 μM) | 10/20 | 109/12 | <0.0001(****) | <0.0001(****) | 0.2424(ns) |
|  | *mcu-1; drp-1* | Empty  Vector | Control | 20/30 | 140/17 | - | - | 0.0003(***) |
|  |  |  | UA (50 μM) | 20/28 | 139/9 |  | 0.2289(ns) | 0.0118(*) |
|  |  | *itr-1*  *(RNAi)* | Control | 12/19 | 110/9 | <0.0001(****) | - | 0.1442(ns) |
|  |  |  | UA (50 μM) | 10/17 | 131/2 | <0.0001(****) | 0.0023(**) | 0.0226(*) |
| 2^◊^ | N2 | Empty  Vector | Control | 17/27 | 113/10 | - | - | - |
|  |  |  | UA (50 μM) | 20/28 | 106/2 |  | 0.0012(**) |  |
|  |  | *itr-1*  *(RNAi)* | Control | 11/18 | 59/12 | <0.0001(****) | - |  |
|  |  |  | UA (50 μM) | 12/16 | 86/14 | <0.0001(****) | 0.8433(ns) |  |
|  | *mcu-1*  *(ju1154)* | Empty  Vector | Control | 18/24 | 110/14 | - | - | 0.6261(ns) |
|  |  |  | UA (50 μM) | 19/26 | 111/0 |  | 0.0054(**) | 0.0560(ns) |
|  |  | *itr-1*  *(RNAi)* | Control | 9/16 | 33/6 | <0.0001(****) | - | 0.0224(*) |
|  |  |  | UA (50 μM) | 10/13 | 57/8 | <0.0001(****) | 0.5618(ns) | <0.0001(****) |
|  | *drp-1* | Empty  Vector | Control | 17/26 | 91/8 | - | - | 0.5906(ns) |
|  |  |  | UA (50 μM) | 16/26 | 107/10 |  | 0.5128(ns) | <0.0001(****) |
|  |  | *itr-1*  *(RNAi)* | Control | 13/23 | 73/14 | 0.0001(***) | - | 0.0039(**) |
|  |  |  | UA (50 μM) | 10/15 | 76/15 | <0.0001(****) | <0.0001(****) | 0.0002(***) |
|  | *mcu-1; drp-1* | Empty  Vector | Control | 21/30 | 104/15 | - | - | 0.0011(**) |
|  |  |  | UA (50 μM) | 20/29 | 109/9 |  | 0.9578(ns) | 0.4031(ns) |
|  |  | *itr-1*  *(RNAi)* | Control | 13/23 | 77/8 | <0.0001(****) | - | 0.0047(**) |
|  |  |  | UA (50 μM) | 10/14 | 66/1 | <0.0001(****) | <0.0001(****) | <0.0001(****) |
| 3^◊^ | N2 | Empty  Vector | Control | 16/24 | 142/10 | - | - | - |
|  |  |  | UA (50 μM) | 19/26 | 140/6 |  | <0.0001(****) |  |
|  |  | *skn-1*  *(RNAi)* | Control | 17/24 | 149/5 | 0.2016(ns) | - |  |
|  |  |  | UA (50 μM) | 16/21 | 142/5 | <0.0001(****) | 0.019(**) |  |
|  | *unc-43* | Empty  Vector | Control | 14/20 | 71/6 | - | - | 0.0088(**) |
|  |  |  | UA (50 μM) | 14/23 | 144/13 | - | 0.8126(ns) | <0.0001(****) |
|  |  | *skn-1*  *(RNAi)* | Control | 13/17 | 136/8 | <0.0001(****) | - | <0.0001(****) |
|  |  |  | UA (50 μM) | 13/16 | 145/5 | <0.0001(****) | 0.3576(ns) | <0.0001(****) |
| 4^◊^ | N2 | Empty  Vector | Control | 16/24 | 132/9 | - | - | - |
|  |  |  | UA (50 μM) | 19/27 | 146/3 | - | <0.0001(****) |  |
|  |  | *skn-1*  *(RNAi)* | Control | 16/21 | 146/4 | 0.0222(*) | - |  |
|  |  |  | UA (50 μM) | 16/21 | 142/0 | <0.0001(****) | 0.9449(ns) |  |
|  | *unc-43* | Empty  Vector | Control | 14/22 | 119/30 | - | - | 0.0112(*) |
|  |  |  | UA (50 μM) | 14/22 | 139/17 | - | 0.7230(ns) | <0.0001(****) |
|  |  | *skn-1*  *(RNAi)* | Control | 13/18 | 139/9 | 0.0002(***) | - | <0.0001(****) |
|  |  |  | UA (50 μM) | 13/16 | 146/3 | <0.0001(****) | 0.0140(*) | <0.0001(****) |
| 5^◊^ | N2 | Empty  Vector | Control | 16/24 | 137/29 | - | - | - |
|  |  |  | UA (50 μM) | 18/27 | 134/47 | - | 0.0003(***) |  |
|  |  | *emc-3*  *(RNAi)* | Control | 16/24 | 140/40 | 0.1847(ns) | - |  |
|  |  |  | UA (50 μM) | 16/23 | 157/16 | <0.0001(****) | 0.0941(ns) |  |
|  |  | *tmco-1*  *(RNAi)* | Control | 16/23 | 142/54 | 0.7955(ns) | - |  |
|  |  |  | UA (50 μM) | 15/23 | 146/25 | <0.0001(****) | 0.3904(ns) |  |
| All experiments were carried out at 20°C in NGM plates seeded with HT115 transformed with the indicated RNAi plasmid construct and supplemented with 2 mM IPTG.  *p<0.05, **p<0.01, ***p<0.001, ****p<0.0001 - Log-rank (Mantel-Cox) test  ^1^T = total/C = censored  ^◊^marks data shown in figures | | | | | | | | |

**Supplementary Data**

**Figure S1.** Urolithin A triggers widespread alterations in organellar architecture, transcriptome and proteome. (**A**-**C**) Representative images of 1-day- and 8-days-old nematodes expressing in their intestine an ER-localized CemOrange2 (*nhx-2* promoter) (**A**), a mitochondria-targeted mKate2 (*ges-1* promoter) (**B**), or a lysosomal CemOrange2 (*nhx-2* promoter), under control conditions and treated with RNAi against *pink-1*, *pdr-1* or *dct-1*, or supplemented with UA (scale bars: 20 μm). (**D**) Quantification of mitochondrial content of 1-day- and 8-days-old nematodes, as judged by the fluorescence intensity of a mitochondria-targeted mKate2 expressed in body wall muscles (*myo-3* promoter), intestine (*ges-1* promoter) or neurons (*rgef-1* promoter), under control conditions or treated with UA (data presented as mean ± SD; ns P>0.05, *P < 0.01, **P < 0.001, ***P < 0.001, ****P < 0.0001; two-way ANOVA). (**E** and **F**) PCA analyses from transcriptomic (**E**) and proteomic (**F**) results indicate the distribution of samples across two principal components. (**G** and **H**) Volcano plot depicting differentially expressed genes (DEGs) (**G**) and differentially expressed peptides (DEPs) (**H**) in UA-treated *versus* untreated (control) nematodes. (**I** and **J**) Heatmaps depicting transcriptome (**I**) and proteome (**J**) changes in nematodes post-UA treatment; color bars indicate the z-score intensity of the rows. (**K** and **L**) Dot plots illustrating enriched Gene Ontology (GO) terms associated with calcium, endoplasmic reticulum (ER), lysosomal, mitochondrial and peroxisomal function derived from transcriptomic analysis (**K**) or high-resolution proteomic study (**L**). X-axis and dot size represent the number of significant genes/peptides that correspond to each GO term. Dot color reflects statistical significance. (**M**) Map of significantly enriched GO terms representing biological processes or cellular components affected by UA treatment at the proteome level. Nodes represent enriched GO terms and edges their interrelationships, clustered by functional similarity. Node size corresponds to statistical significance. Color opacity represents the number of proteins assigned to each term.

**Figure S2.** UA-mediated calcium elevation induces mitophagy and sustains mitochondrial mass and membrane potential during ageing. (**A-D**) Fluorescence intensity (**A** and **C**) and representative images (**B** and **D**) of 4-days-old transgenic nematodes expressing the calcium biosensor GCaMP6.0 in touch receptor neurons (*mec-7* promoter) (**A** and **B**), or GCaMP2.0 under the ubiquitous promoter *let-858* (**C** and **D**), under control conditions and treated with UA, NMN or PQ, in the presence or absence (H_2_O) of the Ca^2+^-chelating agent EGTA. In the case of mechanosensory neurons, GCaMP6.0 fluorescence was normalized to that of RFP, that is simultaneously expressed under the same promoter (scale bars: 50 μm; data presented as mean ± SD; ns, P >0.05, ***P < 0.001; one-way ANOVA). (**E)** Mitophagic flux estimated by the ratio of GFP and DsRed double-positive:DsRed-only-positive particles in 4 days old transgenic nematodes expressing the double fluorophore mtRosella in body-wall muscles (*myo-3* promoter), under control conditions of treated with UA, PQ, NMN in the presence or absence (H_2_O) of EGTA (data presented as mean ± SD; ns, P >0.05, ***P < 0.001; one-way ANOVA.) (**F-H**) Mitophagic flux estimated by the ratio of GFP and DsRed double-positive:DsRed-only-positive particles (F), mitophagy levels estimated by the GFP:DsRed intensity ratio (**G**) and representative images (**H**) of 4-days-old transgenic nematodes expressing the double fluorophore mtRosella in neurons (*unc-119* promoter), under control conditions or treated with UA or PQ, in the presence or absence (H_2_O) of EGTA. Low ratios of GFP:DsRed intensity correspond to high levels of mitophagy (ns P > 0.05, ***P < 0.001; one-way ANOVA). Scale bars: 50 μm. (**I**) Quantification of mitochondrial content of 1-day- and 8-day-old nematodes, as judged by the fluorescence intensity of a mitochondria-targeted mKate2 expressed ubiquitously (*eft-3* promoter), under control conditions or treated with UA in the presence or absence of EGTA (data presented as mean ± SD; ns P>0.05, *P < 0.01, ***P < 0.001, ****P < 0.0001; two-way ANOVA). (**J**) Quantification of mitochondrial membrane potential in 1-day- and 8-days-old nematodes, as determined by the intensity of TMRE staining, under control conditions or treated with UA in the presence or absence of EGTA (data presented as mean ± SD; ns P>0.05, **P < 0.01, ****P < 0.0001; two-way ANOVA).

**Figure S3.** Urolithin A triggers responses involving mitochondria, lysosomes and the ER, ultimately activating SKN-1/NRF2. (**A** and **B**) Heatmap of mitochondrial (**A**) and lysosomal (**B**) proteins upregulated in UA-treated groups as compared to control groups. Hierarchical clustering reveals distinct expression patterns between treated and untreated conditions, indicating increased expression of specific mitochondrial and lysosomal proteins upon UA treatment. (**C** and **D**) Quantification of lysosomal acidity, determined by the intensity of LysoSensor Green staining (**C**) and representative images of 4-days-old stained animals (**D**) (scale bars: 100 μm; data presented as mean ± SD; ns P>0.05, **P < 0.01, ****P < 0.0001; one-way ANOVA). (**E**) Relative mRNA levels of *itr-1*, *mcu-1*, *sca-1* in 4-days-old nematodes under control conditions or treated with UA (data presented as mean ± SD; ns P>0.05, *P < 0.05; one-way ANOVA). (**F** and **G**) Quantification of fluorescence intensity (**F**) and representative images (**G**) of 4-days-old transgenic nematodes expressing the calcium biosensor GCaMP6.0 in their body wall muscles (*myo-3* promoter), under control conditions and treated with UA, exposed to RNAi against *mcu-1* or *itr-1* (scale bars: 50 μm; data presented as mean ± SD; ns P >0.05, **P < 0.01; two-way ANOVA). (**H** and **I**) Qualitative assessment (**H**) and representative images (**I**) of myofilaments in body wall muscles of aged (8-days-old) nematodes, visualized by the expression of GFP::MYO-3/myosin, under control conditions and treated with UA, in the presence or the absence of EGTA, upon RNAi-mediated silencing of *itr-1* (scale bars: 50 μm; ns P > 0.05, ****P < 0.0001; Chi-square test). (**J**) Heatmap of differentially expressed ER proteins in UA-treated groups compared to control groups. (**K**) Lifespan of WT (N2) nematodes exposed to RNAi against *emc-3* or *tmco-1*, under control conditions and treated with UA (ns P > 0.05, ***P < 0.001; log-rank (Mantel-Cox) test). (**L**) Relative mRNA levels of *emc-3* and *tmco-1* in 4-days-old nematodes under control conditions or treated with UA (data presented as mean ± SD; ns P>0.05; one-way ANOVA). (**M**) Motility of aged (8-days-old) wild type (N2) animals in a drop of isotonic liquid (M9), under control conditions and treated with UA, exposed to RNAi against *emc-3* or *tmco-1* (ns, P > 0.05, **P < 0.01; two-way ANOVA). (**N**-**P**) Representative fluorescent images of transgenic animals expressing the p*_gst-4_*GFP (SKN-1/NRF2 target) (**N**), p*_sod-3_*GFP (DAF-16/FOXO target) (**O**), or the p*_hlh-30_*HLH-30::GFP (TFEB ortholog) (**P**) transgenes following exposure to UA or NMN with or without the presence of EGTA. Scale bars: 500 μm for (**N** and **O**) and 50 μm for (**P**).

**Figure S4.** The mitophagy inducing and geroprotective effect of Urolithin A requires the basal activity of DRP-1. (**A** and **B**) Representative images (**A**) and mitophagy levels estimated by the GFP:DsRed ratio (**B**) of 4-days-old DRP-1 depleted transgenic nematodes expressing the double fluorophore mtRosella in neurons (*unc-119* promoter), under control conditions and treated with UA. Low ratios correspond to high levels of mitophagy (scale bars: 50 μm; data presented as mean ± SD; ns P > 0.05, **P < 0.01; two-way ANOVA). (**C** and **D**) Representative images (**C**) and quantification of fluorescence intensity (**D**) in 4-days-old transgenic nematodes expressing the calcium biosensor GCaMP6.0 in their body wall muscles (*myo-3* promoter), under control conditions and treated with UA, exposed to RNAi against *drp-1* (scale bars: 50 μm; data presented as mean ± SD; ns P >0.05, **P < 0.01; two-way ANOVA). (**E** and **F**) Qualitative assessment of the mitochondrial network in 4-days-old animals expressing a mitochondria-targeted GFP in their body wall muscles (*myo-3* promoter). Individual animals were scored based on the properties of their network (**E**) and the resulting distribution is presented (**F**) (scale bars: 50 μm; ns P>0.05, *P < 0.01, ****P < 0.0001; Chi-square test). (**G** and **H**) Representative images of 8-day-old transgenic animals expressing GFP::MYO-3/myosin under the endogenous *myo-3* promoter, exposed to RNAi against *drp-1(RNAi)*, under control conditions or treated with UA (**G)**. (**H**) Qualitative assessment of myofilaments in body wall muscles of aged (8-day-old) nematodes. Scale bars: 50 μm. (ns P > 0.05, ***P < 0.0001; Chi-square test). (**I**-**K**) Images of blots (**I**) and quantification of DRP-1::GFP in the cytosolic fraction (normalized to ACT2 levels) (**J**) or the mitochondrial fraction (normalized to HSP-60 levels) (**K**) of 1-day-old animals expressing a *drp-1::gfp*  transgene from the endogenous locus (data presented as mean ± SD; ns P >0.05, *P < 0.05; one-way ANOVA). (**L**) Representative images of 1-day old transgenic nematodes co-expressing a DRP-1::GFP translational fusion from the endogenous locus (green) and a mitochondria-targeted mKate2 (magenta) in body wall muscles (*myo-3* promoter) under control conditions or supplemented with UA. Scale bars: 40 μm.

**Figure S5.** UA triggers mitophagy and delays the age-related decline of locomotion activity via SKN-1 and UNC-43. (**A**) Lifespan curves of WT (N2) animals treated with RNAi against *skn-1*, under control conditions or after supplementation with UA (significance is presented in comparison to the respective untreated control; **P < 0.01, ****P < 0.0001; log-rank (Mantel-Cox) test). (**B** and **C**) Fluorescence intensity of transgenic nematodes expressing the p*_gst-4_*GFP transgene reporter under control conditions or supplemented with UA, in the presence or absence (H_2_O) of the antioxidant NAC (**B**); control conditions or supplemented with UA or NMN, in the presence or absence of EGTA **(C)** (data presented as mean ± SD; ns P>0.05, **P < 0.001, ***P < 0.001, ****P < 0.0001; one-way ANOVA in (**B**) and (**C**)). (**D**) Representative images of 1-day old transgenic nematodes co-expressing a DRP-1::GFP translational fusion from the endogenous locus (green) and a mitochondria-targeted mKate2 (magenta) in body wall muscles (*myo-3* promoter), subjected to RNAi against *unc-43* under control conditions or supplemented with UA. Scale bars: 5 μm. (**E-G**) Mitophagy levels estimated by the GFP:DsRed ratio (**E**) and representative images of 4-day-old transgenic nematodes expressing the double fluorophore mtRosella in (**F**) neurons (*unc-119* promoter) and (**G**) body wall muscle cells, under control conditions or treated with UA. Low ratios correspond to high levels of mitophagy (scale bars: 50 μm; data presented as mean ± SD; ns P > 0.05, ***P < 0.001, ****P < 0.0001; two-way ANOVA). (**H** and **I**) Qualitative assessment (**H**) and representative images (**I**) of myofilaments in body wall muscles of aged (8-day-old) nematodes, visualized by the expression of GFP::MYO-3/myosin, under control conditions and treated with UA, exposed to RNAi against *unc-43* (scale bars: 50 μm; ns P > 0.05, ****P < 0.0001; Chi-square test). (**J**) Motility of aged (8-days-old) wild type (N2) animals in a drop of isotonic liquid (M9), under control conditions and treated with UA, exposed to RNAi against *unc-43* (data presented as mean ± SD; ns P > 0.05, ****P < 0.0001; two-way ANOVA).

**Figure S6.** Urolithin’s A effects on mammalian cells. (**A**-**C**) Transcriptomic analysis of UA-treated HT29 human cells. PCA analysis (**A**) indicates the distribution of control and UA-treated samples across two principal components. 187 genes were found significantly (p value < 0.05 and |FC| ≥ 1.2) upregulated and 50 downregulated between control/untreated and UA treated groups (**B**). Numerous enriched Gene Ontology (GO) terms are associated with calcium, endoplasmic reticulum (ER), lysosomal, mitochondrial, and peroxide regions (**C**). X-axis and dot size represent the number of significant genes corresponding to each GO term. Dot color reflects statistical significance. (**D**-**H**) The effect of UA in HUVEC cells. Cell viability upon UA treatment (**D**), enzymatic activity of cathepsins B and L (**E**), endogenous oxidative load (ROS levels) (**F**) and relative mRNA levels of autophagy- (**G**) and oxidative response- (**H**) related genes were assessed at different concentrations (data in (**E** and **F**) represent mean ± SEM; all other data presented as mean ± SD; ns P > 0.05, * P < 0.05, ** P < 0.01, one-way ANOVA). (**I**) Immunoblots of autophagy-related protein LC3B in HUVECs and BJ fibroblasts, under control conditions and treated with UA, in the presence or the absence of EGTA. (**J** and **K**) Quantification of calcium levels in HUVECs, estimated by the intensity of Fluo-4 AM staining (**J**) and representative CLSM viewing (green) (**K**), under control conditions and treated with UA, in the presence or the absence of EGTA and supplementation with the DRP-1 inhibitor Mdivi-1. Nuclei were counterstained with DAPI (blue) (scale bars: 10 μm; data presented as mean ± SD; ns P > 0.05, * P < 0.05, ** P < 0.01, one-way ANOVA). (**L** and **M**) Quantification of calcium levels in HMC3 human microglial cells, determined by the intensity of Calbryte^TM^ 520 staining (**L**) and representative images (**M**) under control conditions or treated with UA in the presence or absence of EGTA (scale bar: 100 μm; data presented as mean ± SD; ns P > 0.05, * P < 0.05, ** P < 0.01, one-way ANOVA). (**N**) Quantification of calcium levels in mouse embryonic fibroblasts (MEFs), determined by the intensity of Calbryte^TM^ 520 staining, under control conditions or treated with UA in the presence or absence of EGTA (data presented as mean ± SD; **** P < 0.0001, one-way ANOVA). (**O**) CLSM viewing of mitochondria stained with MitoTracker Green in BJ and HUVEC cells (green), under control conditions and treated with UA, in the presence or the absence of EGTA. Nuclei were counterstained with DAPI (blue) (scale bars: 10 μm). (**P**) Oxygen consumption rates (Seahorse Analyzer) of HUVECs under control conditions and treated with UA, in the presence or the absence of EGTA (mean of OCR ± SD). (**Q** and **R**) Representative CLSM images (red) (**Q**) and quantification of membrane potential, estimated by the intensity of TMRE staining (**R**) of HUVECs and BJ fibroblasts, under control conditions and treated with UA, in the presence or the absence of EGTA. Nuclei were counterstained with DAPI (blue) (scale bars: 10 μm; data presented as mean ± SD; ns P > 0.05, ** P < 0.01, *** P < 0.001, one-way ANOVA). (**S** and **T**) Representative CLSM images of BJ fibroblasts and HUVECs stained with MitoSOX (red) for the estimation of endogenous oxidative load (ROS levels) (**S**), or LysoSensor Green (green) for the evaluation of lysosomal acidity (**T**), under control conditions and treated with UA, in the presence or the absence of EGTA. Nuclei were counterstained with DAPI (blue) (scale bars: 10 μm). (**U** and **V**) CLSM viewing (**U**) and quantification of mitophagy levels as judged by fluorescence intensity of Dojindo Mtphagy dye (red) (**V**) in HUVECs, under control conditions and treated with UA, in the presence or the absence of EGTA and supplementation with the DRP-1 inhibitor Mdivi-1 or the SERCA inhibitor thapsigargin. Nuclei were counterstained with DAPI (blue) (scale bars: 10 μm; data presented as mean ± SD; ns P > 0.05, ** P < 0.01, two-way ANOVA). (**W**) Relative mRNA levels of CDKN1A/p21 in HUVECs under control conditions and treated with UA, in the presence or the absence of EGTA, with and without H_2_O_2_ (data presented as mean ± SEM; ns P > 0.05, ** P < 0.01, ***P < 0.001; two-way ANOVA).
